# A zebrafish model of *crim1* loss of function has small and misshapen lenses with dysregulated *clic4* and *fgf1b* expression

**DOI:** 10.3389/fcell.2025.1522094

**Published:** 2025-03-06

**Authors:** Tien Le, Stephanie Htun, Manoj Kumar Pandey, Yihui Sun, Albert Frank Magnusen, Ehsan Ullah, Julie Lauzon, Shannon Beres, Chung Lee, Bin Guan, Robert B. Hufnagel, Brian P. Brooks, Sergio E. Baranzini, Anne Slavotinek

**Affiliations:** ^1^ Division of Human Genetics, Cincinnati Children’s Hospital Medical Center, Cincinnati, OH, United States; ^2^ Division of Medical Genetics, Department of Pediatrics, University of California San Francisco, San Francisco, CA, United States; ^3^ Department of Pediatrics, University of Cincinnati College of Medicine, Cincinnati, OH, United States; ^4^ Department of Neurology, Weill Institute for Neurosciences., University of California San Francisco, San Francisco, CA, United States; ^5^ Institute of Human Genetics, University of California San Francisco, San Francisco, CA, United States; ^6^ Ophthalmic Genetics and Visual Function Branch, National Eye Institute, National Institutes of Health, Bethesda, MD, United States; ^7^ Department of Medical Genetics and Pediatrics, Cumming School of Medicine, Alberta Children’s Hospital, University of Calgary, Calgary, AB, Canada; ^8^ Department of Ophthalmology, Stanford University School of Medicine, Stanford, CA, United States; ^9^ Department of Neurology and Neurosciences, Stanford University School of Medicine, Stanford, CA, United States; ^10^ Stanford University Pediatrics/Medical Genetics, Stanford University, Stanford, CA, United States

**Keywords:** cysteine-rich motor neuron 1, zebrafish, lens development, RNA-seq, colobomatous macrophthalmia with microcornea, coloboma, macrophthalmia

## Abstract

**Introduction:**

Heterozygous deletions predicting haploinsufficiency for the Cysteine Rich Motor Neuron 1 (*CRIM1*) gene have been identified in two families with macrophthalmia, colobomatous, with microcornea (MACOM), an autosomal dominant trait. *Crim1* encodes a type I transmembrane protein that is expressed at the cell membrane of lens epithelial and fiber cells at the stage of lens pit formation. Decreased Crim1 expression in the mouse reduced the number of lens epithelial cells and caused defective adhesion between lens epithelial cells and between the epithelial and fiber cells.

**Methods:**

We present three patients with heterozygous deletions and truncating variants predicted to result in haploinsufficiency for *CRIM1* as further evidence for the role of this gene in eye defects, including retinal coloboma, optic pallor, and glaucoma. We used Clustered Regularly Interspaced Short Palindromic Repeats (CRISPR)/Cas9 to make a stable *Danio rerio* model of crim1 deficiency, generating zebrafish that were homozygous for a 2 basepair deletion, c.339_340delCT p.Leu112Leufs*, in *crim1*.

**Results:**

Homozygous, *crim1* ^−/−^ larvae demonstrated smaller eyes and small and misshapen lenses compared to controls, but we did not observe colobomas. Bulk RNA-Seq using dissected eyes from *crim1* ^−/−^ larvae and controls at 72 h post fertilization showed significant downregulation of crim1 and chloride intracellular channel 4 (*clic4*) and upregulation of fibroblast growth factor 1b (*fgf1b*) and complement component 1, q subcomponent (*c1q*), amongst other dysregulated genes.

**Discussion:**

Our work strengthens the association between haploinsufficiency for *CRIM1* and eye defects and characterizes a stable model of *crim1* loss of function for future research.

## Introduction

Cysteine-rich motor neuron 1 (*CRIM1*; also known cysteine-rich transmembrane BMP regulator 1; Online Mendelian Inheritance in Man (OMIM) #606189) encodes a type I transmembrane protein that contains an extracellular N-terminal insulin-like growth factor-binding protein motif, six von Willebrand factor type C domains, four antistasin-like domains, a transmembrane domain, and a cytoplasmic C-terminal of 76 residues (Brastrom et al., 2019). The human gene has four isoforms, with the longest isoform containing 17 coding exons (NM_016441.3) and producing a 1,036 amino acid (Aa) protein. Heterozygous deletions predicted to result in haploinsufficiency for *CRIM1* have been identified in families with eye defects comprising colobomatous macrophthalmia with an increased axial length of the globe, microcornea, and coloboma of the iris, chorioretinal structures, and optic disc (Toker et al., 2003; Beleggia et al., 2015; Haug et al., 2021). In these families, a deletion of approximately 22 kilobases (kb) that included exons 14 through 17 of *CRIM1* was described in a family with microcornea, coloboma of the iris, choroid, retina, and optic disc, increased axial length, myopia, mild cornea plana, and a shallow anterior chamber (Toker et al., 2003; Beleggia et al., 2015). A deletion of approximately 9 kb removing exons 15–17 and the 3′ untranslated region (UTR) of *CRIM1* was reported in a family with iris and chorioretinal colobomas and microcornea (Haug et al., 2021). These eye findings are known as colobomatous macrophthalmia with microcornea syndrome (MACOM; OMIM #602499) and are inherited as an autosomal dominant trait (Toker et al., 2003; Beleggia et al., 2015; Haug et al., 2021). Additional ocular findings in MACOM have included staphyloma and increased intraocular pressure (Bateman and Maumenee, 1984; Toker et al., 2003; Beleggia et al., 2015; Haug et al., 2021). The eye defects have shown variable expressivity, affecting eye laterality, axial length, and the severity of colobomas (Bateman and Maumenee, 1984; Haug et al., 2021). There have been no prior reports of pathogenic variants apart from the deletions resulting in *CRIM1* haploinsufficiency. A duplication including *CRIM1* and additional genes (GRCh37/hg19 2p23.3-21(chr2:24881528-43460021)x3) was noted in ClinVar, but no phenotypic details were provided. The predicted loss-of-function (pLoF) score for *CRIM1* in gnomAD v4.1 (Chen et al., 2024) is 1.

In murine embryos, *Crim1* expression begins throughout the lens placode at embryonic day (E)10.5 and becomes concentrated in the lens epithelial (LE) and lens fiber (LF) cells at E12.5 (Lovicu et al., 2000; Zhang et al., 2016). As the lens fibers continue to mature, *Crim1* is downregulated, but strong expression is maintained in the LE cells and in the developing LF cells of the lens cortex until postnatal life (Lovicu et al., 2000). Within the LE cells, *Crim1* is localized to the cell membranes and cytoplasm (Zhang et al., 2016). *CRIM1* is also expressed in the endoplasmic reticulum and accumulates at cell–cell contacts after the stimulation of human microvascular endothelial cells with lipopolysaccharide (Glienke et al., 2002).

Although complete loss of function for *Crim1* resulted in perinatal lethality in the mouse, there have been several studies examining eye formation in mice that are homozygous (HZ) for *Crim1* hypomorphic or conditional null alleles (summarized in Supplementary Table S1; Pennisi et al., 2007; Wilkinson et al., 2007; Wilkinson et al., 2009; Chiu et al., 2012; Pennisi et al., 2012; Wilkinson et al., 2012; Phua et al., 2013; Beleggia et al., 2015; Tam et al., 2018; Furuichi et al., 2019). Ocular defects resembling MACOM in the mutant mice have included small lenses, microcornea, a restricted aperture of the anterior eye chamber, and a narrow eye without diminished axial diameter (Beleggia et al., 2015). Additional eye findings have included microphthalmia (Chiu et al., 2012; Zhang et al., 2016), retinal dysplasia (Zhang et al., 2016), and aberrant accumulation of an ‘endothelial-like’ cellular mass or hyaloid cells in the posterior vitreous chamber (Pennisi et al., 2007; Tam et al., 2018). Small and misshapen lenses (Pennisi et al., 2007; Fan et al., 2014; Beleggia et al., 2015; Zhang et al., 2016), congenital cataracts (Zhang et al., 2016), reduced numbers of LE cells with disorganized epithelial cell adhesion, and deficient development of the LF cell mass have also been noted (Beleggia et al., 2015; Zhang et al., 2016; Tam et al., 2018). *Crim1* is critical for the maintenance of the lens epithelium, which becomes anteriorly restricted in the murine mutants due to the early differentiation of LE cells into LF cells (Tam et al., 2018).

Zebrafish have a single, *crim1* transcript that encodes a protein of 1,027 amino acids with 69% identity to human *CRIM1* and a conserved domain structure resembling the human protein (Kolle et al., 2000; Kolle et al., 2003; Kinna et al., 2006). *crim1* expression was observed from early blastula up to 48 h post-fertilization (hpf), consistent with maternal and zygotic expression (Kinna et al., 2006). The expression of *crim1* was first observed in a diffuse pattern along the entire anterior–posterior axis and the notochord (Kinna et al., 2006). *crim1* was also noted in the brain, the intermediate cell mass (ICM), and otic vesicles (Kinna et al., 2006). Using Daniocell, expression in the eye is the strongest after 72 hpf and is predominantly observed in the anterior retinal pigment epithelium (RPE), retinal interneurons, and developing RPE (Farrell et al., 2018; Sur et al., 2023). Prior studies using antisense morpholino (MO) injections targeting *crim1* resulted in embryos with small eyes, small heads, abnormal somite patterning, curved bodies, and an expansion of the ICM compared to controls (Supplementary Table S2; Kinna et al., 2006). The embryos also had an aberrant formation of the intersegmental vessels and dorsal longitudinal anastomotic vessels, implicating *crim1* in vascular development (Kinna et al., 2006). A subsequent study that used a low (1.0–1.5 ng) dose of antisense MO injections revealed increased nuclei clustered in the medial portion of the lens with mild, nuclei-in-lens cataracts but preserved visual attentiveness (Brastrom et al., 2019). Injected larvae were otherwise normal (Brastrom et al., 2019). Higher (2.5–3.0 ng) doses of antisense MOs led to increased numbers of nuclei in the lenses, microphthalmia, and defective retinal lamination (Brastrom et al., 2019).

In this study, we present the clinical details pertaining to three unpublished patients from two families with ocular defects due to pathogenic variants predicting haploinsufficiency for *CRIM1*. To better understand the etiology of the eye defects associated with decreased *CRIM1* expression, we used Clustered Regularly Interspaced Short Palindromic Repeats (CRISPR)/Cas9 to generate a stable zebrafish model of reduced *crim1* function with smaller eyes and lenses. We describe the phenotype of the *crim1* ^*−/−*^ mutant larvae and provide bulk RNA-seq data to support the role of this gene in eye development.

## Materials and methods

### Patients with pathogenic variants predicting haploinsufficiency for *CRIM1*

Patients with *CRIM1* deletions and pathogenic variants were ascertained by GeneMatcher (Sobreira et al., 2015). Phenotypic and genotypic details were collected in a spreadsheet. Consent was obtained for the publication of anonymous clinical details for each participant.

### Generation and phenotyping of *crim1* ^−/−^ larvae

All animal experiments were performed under protocols approved by the Institute for Animal Care and Use Committee (IACUC) at the University of California, San Francisco, and the IACUC committee at Cincinnati Children’s Hospital Medical Center (IACUC #2022-0040). EKW strain zebrafish were maintained at 28°C on a 14-h (h) light/10-h dark cycle. We used a modification of the previous CRISPR methodology to target the single *crim1* gene (ENSDART00000050534.6) in *Danio rerio* (Talbot and Amacher, 2014). We designed a small guide (sg)RNA targeting *crim1* prior to the coding sequence for the von Willebrand factor type C domains, antistasin-like domains, and the cysteine-rich motor neuron 1 protein domain (Supplementary Table S3). We injected zebrafish eggs at the one-cell stage and used Sanger sequencing in batches of 10–20 larvae at 48 hpf to assess the success of the injections. F0 larvae with insertion/deletion (indel) variants were raised and genotyped at 6–8 weeks by fin clipping after inducing anesthesia by immersing the fish in 0.02% tricaine methanesulfonate (FINQUEL or Tricaine-S) until gill movement was slowed. DNA was extracted from the clipped tail fin and Sanger-sequenced to assess successful gene targeting (for genotyping primers, see Supplementary Table S4). We selected an F0 founder with a 2 base pair (bp) deletion, c.339_340delCT p.Leu112Leufs*3 that introduced a frameshift and predicted premature protein truncation and nonsense-mediated decay for the *crim1* transcript (for Sanger sequencing traces, see Supplementary Figure S1). After outcrossing this F0 founder, F1 progeny were raised and genotyped using DNA obtained from fin-clipping, as mentioned above. Heterozygous F1 adults were in-crossed to obtain F2 zebrafish that were homozygous (HZ) for the 2 bp deletion in *crim1*. These HZ mutant larvae, henceforth referred to as *crim1* ^*−/−*^ larvae, were viable. We raised and bred *crim1* ^*−/−*^ larvae that appeared normal, without any eye defects, for our experiments. We also examined eye morphology in heterozygous *crim1* ^+/−^ larvae. Our control fish were derived from the EKW strain or the NBT:DsRed strain that labels neurons under the neural ß-tubulin promoter and is not known to have any eye defects (Mazzolini et al., 2018).

We examined the *crim1* ^*−/−*^ larvae, *crim1* ^*+/−*^ larvae, and controls at 1–6 days post fertilization (dpf) for the ocular and lens findings previously observed in patients with *MACOM* and prior models of *Crim1* and *crim1* loss of function. We measured eye and lens size, head size, and body length compared to control larvae of the same age and developmental stage. Larvae with bent tails were measured along the body axis and the curved tail to capture the full length of the larva. We examined eye and lens morphology with hematoxylin and eosin (H&E) staining and immunohistochemistry (IHC) and used light microscopy to visualize the LE cells, LF cells, and retinal cell layers according to previously described methods (Krall et al., 2018; see Supplementary Table S4 for antibodies used and Supplementary Data for Method). Larvae photographed with differential interference contrast (DIC) microscopy at 3 dpf were treated with propylthiouracil after fertilization according to standard methods (Elsalini and Rohr, 2003). We used rabbit anti-phospho-histone H3 at 1–400 dilution (PH3; #9701S, Cell Signaling, Danvers, MA) with anti-rabbit Alexa Fluor 488 (Invitrogen, Grand Island, New York) for PH3 staining. We scored two sections per eye for PH3+ cells. At 48 hpf, we scored 7 control larvae, 5 *crim1* ^*+/−*^ larvae, and 4 *crim1* ^*−/−*^ larvae. At 72 hpf, we scored 9 control larvae, 4 *crim1* ^*+/−*^ larvae, and 8 *crim1* ^*−/−*^ larvae.

### 5-Bromo-2′-deoxyuridine labeling and scoring

For BrdU labeling, 48 hpf and 72 hpf larvae were incubated in egg water containing 10 mM 5-bromo-2′-deoxyuridine (BrdU)/15% dimethyl sulfoxide (DMSO) solution (Sigma-Aldrich, St. Louis, MO) on ice for 30 min. Post-incubation, larvae were rinsed with fresh egg water and then placed at 28°C for 1 h (Janssens et al., 2013). BrdU-labeled larvae were euthanized and fixed with 4% paraformaldehyde in phosphate-buffered saline (PBS). BrdU-labeled sections were treated with 2N hydrochloric acid (HCl)/0.5% Triton/PBS for 60 min prior to incubation with mouse anti-BrdU (#B2531, 1-400, Sigma-Aldrich), followed by conjugation with anti-mouse Alexa 488 (Invitrogen, Carlsbad, CA). At least two sections per eye and both eyes were counted manually for BrdU + cells using ImageJ software.

### ‘Bulk’ RNA-seq in *crim1* ^−/−^ mutant larvae

We performed ‘bulk’ RNA-seq experiments in dissected eyes from in-crossed *crim1* ^*−/−*^ larvae and controls at 72 hpf according to the previously described methods (Krall et al., 2018). RNA quality and quantity were reviewed using a bioanalyzer (2100 Bioanalyzer, Agilent), and libraries were prepared with an Ovation^®^ RNA-Seq System V2 Kit (NuGEN). With this method, total RNA was reverse transcribed to synthesize first-strand cDNA using a combination of random hexamers and a poly-T chimeric primer (Krall et al., 2018). The RNA template was then partially degraded by heating, and the second-strand cDNA was synthesized using DNA polymerase. The double-stranded DNA was then amplified using single primer isothermal amplification (SPIA). SPIA is a linear cDNA amplification process in which RNase H degrades RNA in DNA/RNA heteroduplex at the 5′-end of the double-stranded DNA, after which the SPIA primer binds to the cDNA and the polymerase starts replication at the 3′-end of the primer by displacement of the existing forward strand (Krall et al., 2018). Random hexamers were then used to linearly amplify the second-strand cDNA. Finally, libraries from the SPIA-amplified cDNA were prepared using the Ultralow DR Library Kit (NuGEN). The RNA-seq libraries were analyzed for quality and primer dimers using a bioanalyzer and quantified by quantitative polymerase chain reaction (qPCR) prior to sequencing (KAPA Library Quantification Kit). High-throughput sequencing was conducted using single-end 50 lane(s) on a HiSeq 4000 instrument (Illumina).

The quality of raw single-end reads of the RNA-seq dataset was tested by FastQC (http://www.bioinformatics.babraham.ac.uk/projects/fastqc/) to evaluate the per-base sequence quality, quality scores, sequence length distribution, and overrepresented adapter/primer sequences. After quality control with FastQC, data trimming and clipping were performed using BBDuk and aligned against the zebrafish reference genome (GRCz11) using STAR. Alignment results were then assessed to check the quality with Qualimap. RSEM was used to quantify genes. Finally, DESeq2 was used to conduct a downstream analysis for differentially expressed genes. Statistically significant differentially expressed genes (DEGs) with log2 (fold change) greater than ±0.5, an adjusted p-value ≤0.05, and the mean fragments per kilobase of exon per million fragments mapped (FPKM) ≥1 in all datasets were identified by comparing *crim1* ^−/−^ mutants with control datasets using an in-house Python script. Principal component analysis (PCA) was performed on global gene expression to determine the relatedness between the individual replicates of wildtype and mutant datasets.

### Reverse transcriptase quantitative polymerase chain reaction

We designed gene-specific, reverse transcriptase quantitative polymerase chain reaction (RT-qPCR) primers flanking at least one intron for the top-ranking up- and down-dysregulated genes in the bulk RNA-seq experiments (for primers, see Supplementary Table S5). Each RT-qPCR was run in triplicate with melt curve analysis showing a single peak. All experiments were performed on at least three biological replicates of control and *crim1* ^*−/−*^ larvae, following the previously described methods (Krall et al., 2018). We generated a time course of *crim1* expression from 24 to 72 hpf. Eyes were dissected from zebrafish larvae into cold, 1x PBS at 72 hpf. A total of 40 pairs of eyes were pooled in a 1.5-mL microfuge tube, spun, and snap-frozen at −80°C after PBS removal. Total RNA was extracted using the PureLink RNA Mini Kit (Invitrogen, Carlsbad, CA). RNA was treated with Ambion™ DNase I (RNase-free, Invitrogen, Carlsbad, CA) to remove impurities. cDNA was synthesized from 1.5 µg of total RNA with oligo(dT)_20_ priming using SuperScript III Reverse Transcriptase (Invitrogen, Carlsbad, CA). Reactions were run on an ABI Prism 7500 Sequence Detection System, and data were analyzed according to the ΔΔCt method using *eef1a1l1* (NM_131263.1) as an internal control according to previously described methods (Wu et al., 2015).

### Terminal deoxynucleotidyl transferase dUTP nick end labeling assay

Terminal deoxynucleotidyl transferase dUTP nick end labeling (TUNEL) staining was performed on cryosections of controls and *crim* ^−/−^ larvae from 48 to 72 hpf, according to the manufacturer’s protocol (1168479510, Roche, Indianapolis, IN). We examined a minimum of four larvae from each experimental group and analyzed 2–4 non-consecutive mid-eye sections per eye at each timepoint. For apoptotic cleaved caspase-3, we stained cryosections from controls and *crim* ^−/−^ larvae at 24, 30, 48, and 72 hpf. A minimum of four larvae from each experimental group were analyzed using 2–4 non-consecutive sections from the middle of each eye for each timepoint. Sections were stained and counted using the count tool in ImageJ software. The number of TUNEL-positive or cleaved caspase-3-positive cells was divided by the total number of nuclei labeled with 4′,6-diamidino-2phenylindole (DAPI) and plotted against age. After calculating the mean ± standard error (SE), groups with statistically significant differences compared to controls were determined using a Student’s t-test.

### Enzyme-linked immunosorbent assay

Fifty whole larvae were collected from controls and *crim1* ^−/−^ larvae at 12 and 24 hpf. Additionally, 100 whole eyes were collected from controls and *crim1* ^−/−^ larvae at 48 and 72 hpf. These eye tissues were rapidly frozen on dry ice, then thawed, and mechanically homogenized. The homogenates were centrifuged at 8,000 relative centrifugal force (rcf) at 4°C. The resulting supernatants containing soluble proteins were carefully collected. These supernatants were then used for quantifying C1q levels using an enzyme-linked immunosorbent assay (ELISA), following the protocols provided by the manufacturer (MBS023066, MyBioSource, San Diego, CA). Optical density at 450 nm was read using a Bio-Tek Microplate Reader System with Gen5 software (Bio-Tek, Santa Clara, CA).

### Immunoprecipitation

Fifty heads were collected from control and *crim1* ^−/−^ larvae at 72 hpf. Whole heads were homogenized, solubilized, and washed (Michel and Miller, 2023). Supernatants were pre-cleared, and the protein lysates were measured using a Direct Detect Spectrometer system (DDHW00010, Millipore Sigma, Burlington, MA, United States). Lysates were incubated with recombinant anti-Crim1 (AB5699, 1:200, Sigma-Aldrich, St. Louis, MO) and anti*-β1-*integrin (Itgβ1; clone MB1.2, MAB1997, 1:50, Sigma-Aldrich, St. Louis, MO) at 2 µg of antibody overnight at 4°C. After washing, the supernatants were used for Western blot analysis according to standard methods. We loaded 20 µL of 40 µg of immunoprecipitants and 20 µL of 4 µg of input using 4%–20% Mini-PROTEAN^®^ TGX Stain-Free™ Protein Gels (Bio-Rad, Hercules, CA).

### Statistics

Statistical analysis was performed using t-tests according to standard methods. We used a significance level of *P-*value < 0.05.

## Results

### Patients with pathogenic variants predicting haploinsufficiency for *CRIM1* demonstrate coloboma and optic atrophy

The clinical and genetic data from patients with *CRIM1* variants predicting haploinsufficiency are summarized in Table 1 and presented in full in Supplementary Table S6. We ascertained a 48-year-old female with bilateral cornea plana and microcornea, bilateral cataracts (right eye, dense nuclear and partially subluxated; left eye, surgically removed intraocular lens, aphakic), bilateral iris colobomas, and a coloboma of the retina in the left eye, with bilateral retinal detachments. Due to significant corneal band keratopathy and dense media opacity in the right eye, examination of the optic nerve and posterior pole of the retina could not to be performed. Her aphakic left eye distance visual acuity was 20/80 with a +7.00 + 2.00 × 81 refraction. Her right eye had no light perception. Other findings included mild, low-frequency sensorineural hearing loss that normalized in the mid frequencies and sloped to a mild-to-moderate loss at high frequencies bilaterally. A renal ultrasound (US) scan showed mild, focal caliectasis/hydronephrosis of the right kidney at the mid to lower pole of the collecting system and diffuse mild left hydronephrosis. The patient and her father had a heterozygous, 39,942 bp deletion encompassing exons 14 to 17 and the 3′ UTR region of *CRIM1*. The deletion also included exons 6 to 8 of Fasciculation and Elongation protein, Zygin/zeta-2 (*FEZ2*; OMIM #62486), which has not yet been associated with a phenotype in humans.

**TABLE 1:** Description of *CRIM1* variants and eye findings in three new patients with *CRIM1* haploinsufficiency.

Reference/patient	Toker et al., 2003 (n = 11)	Haug et al., 2021 (n = 3)	Patient 1	Patient 2	Patient 3
Reference genome	hg19	hg19			hg19
*CRIM1* transcript/genomic position	NM_016441.2; chr2:36,757,668–36,780,274del	g.36769283_36778290del			chr2:g.36761544_36801468del
Nucleotide variant	Deletion of exons 14 through 17 of *CRIM1* with 3′UTR of *FEZ2*	Deletion of exons 14 through 17 of *CRIM1* with 3′UTR of *FEZ2*	c.2701delC:p.Leu901Cys*22	c.2701delC:p.Leu901Cys*22	Deletion of exons 14 to 17 and 3′UTR of *CRIM1* and deletion of exons 6 to 8 of the *FEZ2* gene
Flanking sequence	4-bp^1^ microhomology CTTG	2-bp microhomology CT			4-bp microhomology GAAG
Deletion	22,606 bp deletion	9,008 bp deletion			39,924 bp deletion
Testing	Exome sequencing	Exome sequencing	Exome sequencing	Parental control for patient 1	MAC^2^ Panel, genome sequencing
DEMOGRAPHICS					
Gender	4F^3^; 7 M^4^	F	M	M	F
Age at last examination	5 m^5^–62 y^6^	2 y–75 y	12 y	48 y	48 y
Ethnicity	Turkish	European	South Asian (India)	South Asian (India)	European and Middle Eastern
Family history; inheritance	Y; AD^7^ inheritance	Y; AD inheritance	Y; son of patient 2 AD inheritance	Y; father of patient 1; no other family history; AD inheritance	Y; father of the patient also affected; AD inheritance
Consanguinity	No	Not known	No	No	No
EYES					
Microcornea	Yes; all <9 mm	Yes; unilateral 2/3	No (exam at 14 y)		Yes, OS^8^>OD^9^
Coloboma (iris/choroid/ret./OD)	Yes; all iris, choroid, retina, and OD in all but two	Yes; 3/3 iris, choroid, retina	No (exam at 14 y)		Iris bilaterally; OD nerve and retina difficult due to dense media opacity. OS retina but not optic nerve
Cornea plana	Yes; all flatter corneal curvature		No		Yes, OS > OD
Myopia	Yes; all −0.50 to −22.00 diopters		Yes; wears contacts		No
Shallow anterior chamber			No		No
Axial length elongation	Yes; 23.0–27.2 mm		Axial length very long for age; axial length OD: 25.80 mm; OS: 26.26 mm (12 y)		Unknown
Nystagmus	Yes; rotary/horizontal in 9/11		No		No
Strabismus	Yes; 8/11; 4 exotropia and 4 esotropia				No
Cataracts	No		No		Yes OU^10^. Was aphakic OS at exam
Astigmatism	Yes; with myopia				Yes; OD
Fundus	All with retinal pigment epithelial atrophy, most with macular atrophic changes		Neuro ophthalmology exam at 14 y. Right disc, cupped, very thin superiorly, 3+ pallor diffusely, cup/disc ratio 0.6, normal macula, normal vessels. Left disc, normal, pallor at neuro retinal rim temporally, cup/disc ratio 0.8, normal macula, normal vessels		OD: poorly evaluated due to media opacity, atrophic areas of retina; OS; tigroid fundus. Inferior coloboma, diffuse atrophy in the posterior pole along with patches of atrophy (peripapillary, inferior to optic nerve, and superotemporal, to the fovea)

bp^1^, basepair; MAC^2^, microphthalmia, anophthalmia, coloboma; F^3^, female; M^4^, male; m^5^, months; y^6^, years AD^7^, autosomal dominant; OS^8^, left eye; OD^9^, right eye; OU^10^, bilateral eyes.

A second family of two generations comprised a 12-year-old male with myopia and his father. The boy had an increased axial length and vision loss from optic atrophy in both eyes, with temporal field loss in the right eye and left total inferior temporal field loss. The optic nerves were cupped with 3+ optic atrophy of the neuro-retinal rim in the right eye. The left optic disc was normal, with pallor at the temporal neuro-retinal rim. Optical coherence tomography (OCT) showed a severely thin retinal nerve fiber layer in his right eye and a mildly thin retinal nerve fiber layer in his left eye. Other medical findings comprised febrile seizures at 2 years of age, testicular torsion treated with orchidopexy, an arachnoid cyst, and a Chiari malformation that reportedly resolved on subsequent magnetic resonance imaging (MRI) of the brain. His milestones and development were normal, except for requiring 6 months of speech therapy at the age of 2 years. His father was myopic and wore glasses from 10 years of age and was diagnosed with corneal pannus, cataract at a young age, and visual field loss. The father’s examination showed a similar optic nerve appearance to his son and bilateral, diffuse optic atrophy with cupping. Both father and son had a single-nucleotide deletion in *CRIM1*, c.2701delC p.Leu901Cysfs*22 (NM_016441.3). This variant affects exon 15 out of 17 exons in the longest isoform of *CRIM1*, which is the only known coding transcript. The variant is not present in the 1000 Genomes Project but was found in one individual in gnomAD v4.1 (1/1,424,618 alleles for an allele frequency of 7.02e^−7^; no homozygous allele) and predicts premature protein truncation and nonsense-mediated decay (MutationTaster; Schwarz et al., 2010). The nucleotide variant also increases an acceptor splice site (wildtype 0.21 mutant 0.27; exon gtgg/CCCA intron; MutationTaster; Schwarz et al., 2010).

### *crim1* ^−/−^ mutant larvae demonstrate microphthalmia and lens defects

**FIGURE 1:**
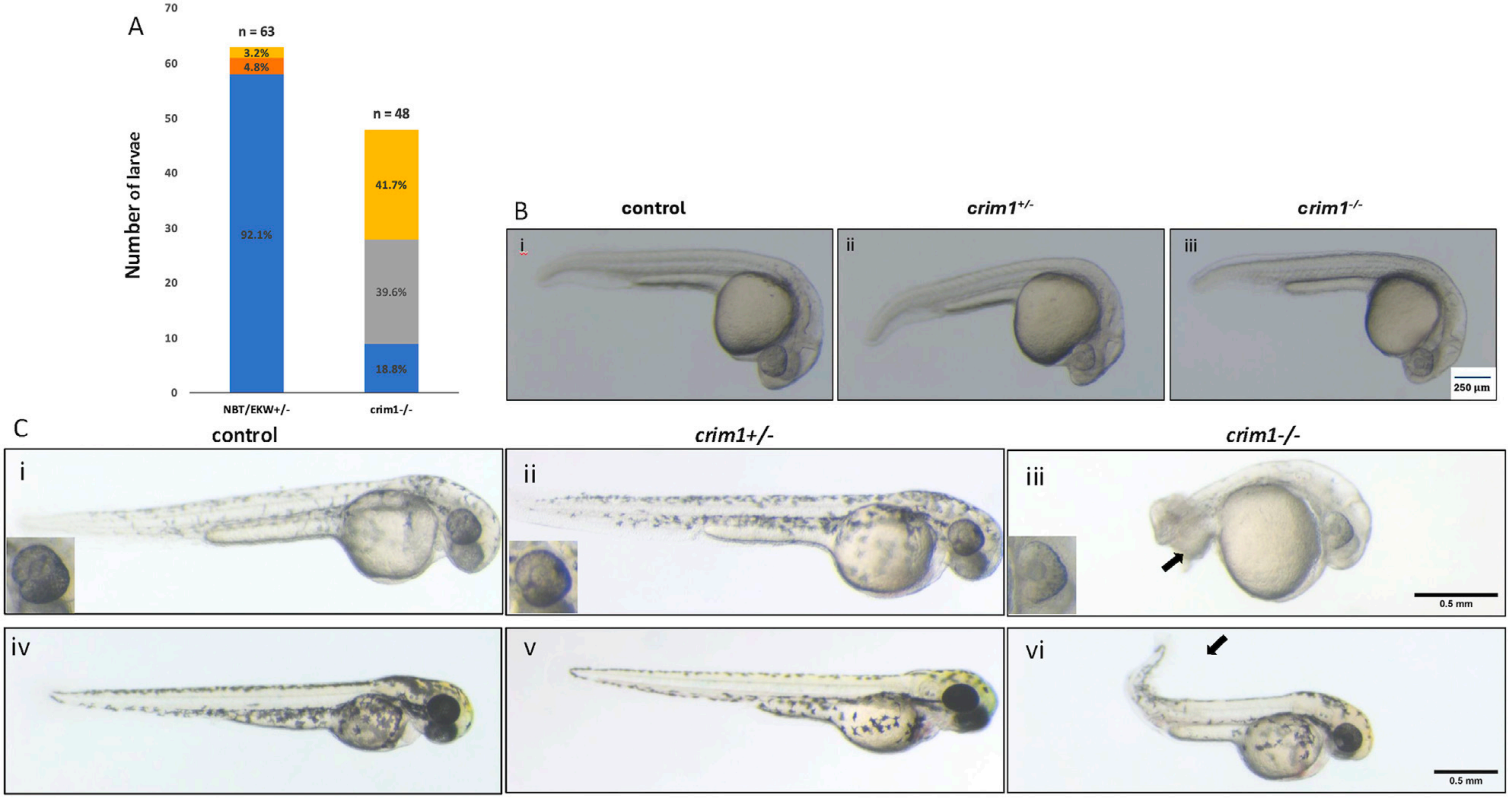
Morphology and appearance of control, heterozygous, *crim1* ^+/−^, and *crim1* ^−/−^ larvae that are homozygous for a 2 base pair deletion, c.339_340delCT p.Leu112Leufs*3 at 24, 48, and 72 h post fertilization (hpf). **(A)** Morphology for control (n = 63) and *crim1* ^−/−^ larvae (n = 48) that are homozygous for a 2 base pair deletion in *crim1*. The columns are colored according to the numbers and percentages of larvae with a normal phenotype (blue), mild deformity (orange), curved body axis (yellow), and malformations such as edema, cardiac defects, and severely shortened body (gray). The *crim1* ^−/−^ larvae have an increased rate of abnormalities compared to controls. **(B)** Representative photographs of control larvae (panel i), heterozygous *crim1* ^+/−^ larvae (panel ii), and homozygous *crim1* ^−/−^ larvae (panel iii) at 24 hpf. The heterozygous *crim1* ^+/−^ larvae and homozygous *crim1* ^−/−^ larvae appear similar to the control. **(C)** Representative photographs of control larvae (panels i and iv), heterozygous *crim1* ^+/−^ larvae (panels ii and v), and homozygous *crim1* ^−/−^ larvae (panels iii and vi) at approximately 38 hpf (panels i, ii, and iii) and 72 hpf (panels iv, v, and vi). The heterozygous *crim1* ^+/−^ larvae appear similar to controls, but the homozygous *crim1* ^−/−^ larvae show shortened body axes and curved tails at both time periods, as indicated by the arrows. Scale bars = 500 µm.

We in-crossed male and female zebrafish that were HZ for the 2bp deletion in *crim1*. At 48 hpf, the *crim1* ^−/−^ larvae showed increased numbers of malformations and shortened body lengths compared to controls (Figure 1A). Curved and shortened body axes and shortened or deformed tails were observed in 81.3% of *crim1* ^−/−^ larvae compared to 3.2% of controls (Figure 1A). At 24 hpf, there were no obvious external differences between the controls, heterozygous *crim1* ^+/−^ larvae, and *crim1* ^−/−^ larvae (Figure 1B). However, by 48 hpf, the *crim1* ^−/−^ mutants demonstrated tail deformities (Figure 1C, panel iii), and at 72 hpf, the *crim1* ^−/−^ mutants had curved tails similar to prior MO models of *crim1* loss of function (Figure 1C, panel vi; Kinna et al., 2006). Heterozygotes appeared similar to controls at 48 hpf (Figure 1C, panel ii) and 72 hpf (Figure 1C, panel v), and for this reason, we did not quantify the malformations or study the heterozygotes further. Approximately 18% of the HZ *crim1* ^−/−^ larvae retained a normal external appearance and ocular appearance (Figure 1A).

**FIGURE 2:**
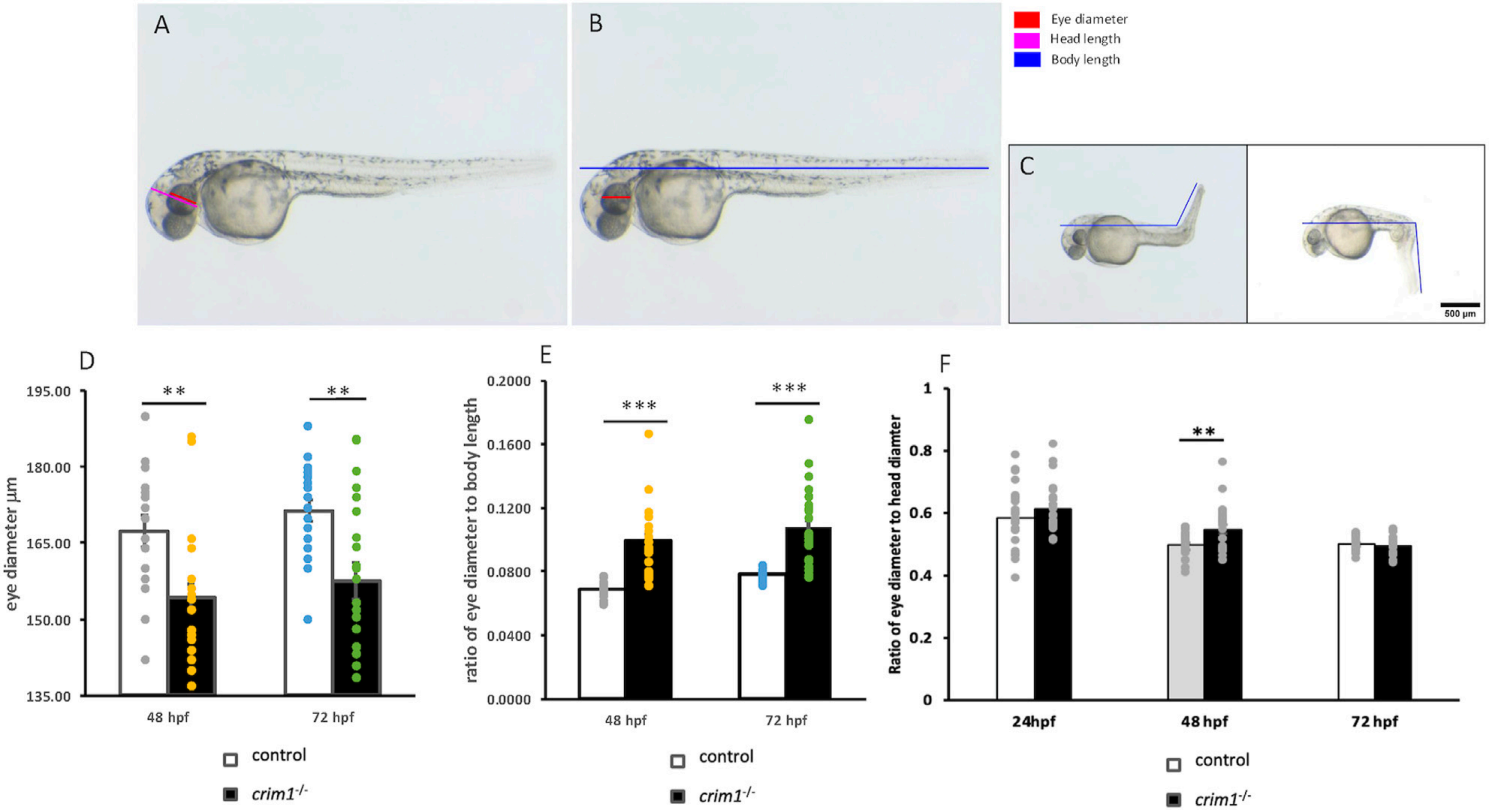
Eye, head, and body measurements from control and *crim1* ^−/−^ larvae that are homozygous for a 2 base pair deletion, c.339_340delCT p.Leu112Leufs*3, at 48 and 72 h post fertilization (hpf). **(A)** Control larva showing measurements obtained for eye diameter and head length. **(B, C)** Control larva showing site of measurements for eye diameter and body and tail length. **(D)** Measurements of eye diameter in control larvae (white columns) and *crim1* ^−/−^ larvae (black columns) at 48 hpf and 72 hpf. Each dot represents a single measurement. Eye diameter is significantly decreased in the *crim1* ^−/−^ larvae compared to controls. **(E)** Ratio of eye diameter to body length in control larvae (white columns) and *crim1* ^−/−^ larvae (black columns) at 48 hpf and 72 hpf. Each dot represents a single measurement. The *crim1* ^−/−^ larvae demonstrate a significantly increased ratio for eye diameter to body length compared to controls. **(F)** Ratio of eye diameter to head diameter in control and *crim1* ^−/−^ larvae. Each gray dot represents a measurement at 24 hpf, 48 hpf, and 72 hpf. The height of the white columns represents the mean of the ratio of eye diameter to head diameter for control larvae and the height of the black columns representing the mean of the ratio of eye diameter to head diameter for the *crim1* ^−/−^ larvae. The *crim1* ^−/−^ larvae show a significantly increased ratio of eye diameter to head diameter at 48 hpf (*P* = 0.008), but the ratio was not significantly increased at 24 hpf or 72 hpf.

We measured the longest axis for eye diameter, head diameter, and body length in the *crim1* ^−/−^ larvae and controls (see Figures 2A–C for measurements obtained). We found a significant decrease in the eye size in the *crim1* ^−/−^ larvae at 48 hpf and 72 hpf (Figure 2D; *P-*value for 48 hpf = 0.003 and *P-*value for 72 hpf = 0.002). This decrease in the eye size resembles microphthalmia observed in the mouse models of *Crim1* loss of function (Supplementary Table S1; Chiu et al., 2012; Fan et al., 2014; Zhang et al., 2016). In contrast, the ratio of eye diameter to body length was significantly increased in the *crim1* ^−/−^ larvae at 48 hpf and 72 hpf (*P*-value for 48 hpf = 1.71e^−06^ and *P*-value for 72 hpf = 2.65e^−05^), consistent with a greater reduction in the body length than in the eye size (Figure 2E). Finally, the ratio of eye diameter to head diameter was increased in the *crim1* ^−/−^ larvae compared to controls at 48 hpf but not at 24 hpf or 72 hpf (Figure 2F).

**FIGURE 3:**
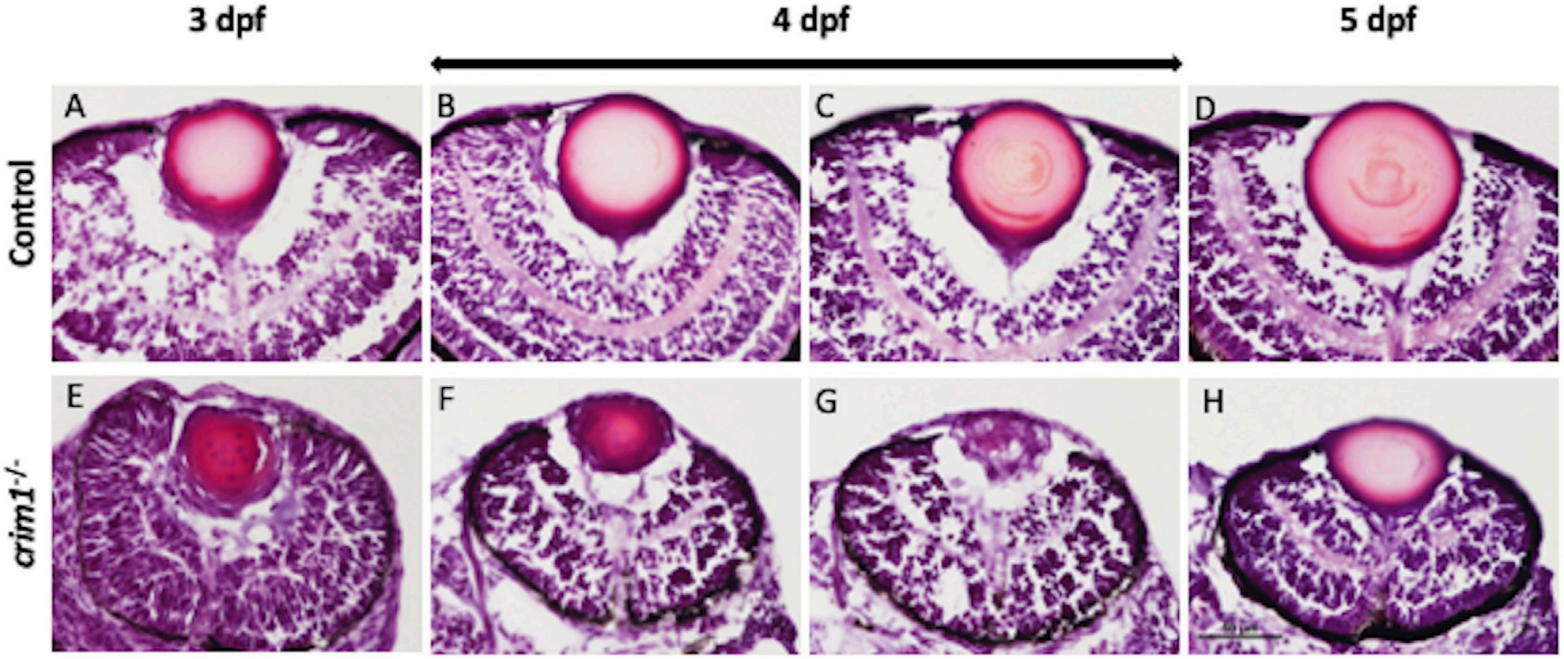
Representative images of zebrafish eyes stained with H&E from control and *crim1* ^−/−^ larvae that are homozygous for a 2 base pair deletion, c.339_340delCT p.Leu112Leufs*3, at 3–5 days post fertilization (dpf). The top row shows control larvae at 3 dpf **(A)**, 4 dpf **(B, C)**, and 5 dpf **(D)**, showing normal lens and eye development. The bottom row shows homozygous *crim1* ^−/−^ larvae at 3 dpf **(E)**, 4 dpf **(F, G)**, and 5 dpf **(H)**, showing reduced lens and eye size in the *crim1* ^−/−^ lenses at 3 and 4 dpf **(E, F)**. At 3 dpf, 6–8 larvae were scored, and at 4 and 5 dpf, 10–12 larvae were scored. Scale bar = 50 µm.

**FIGURE 4:**
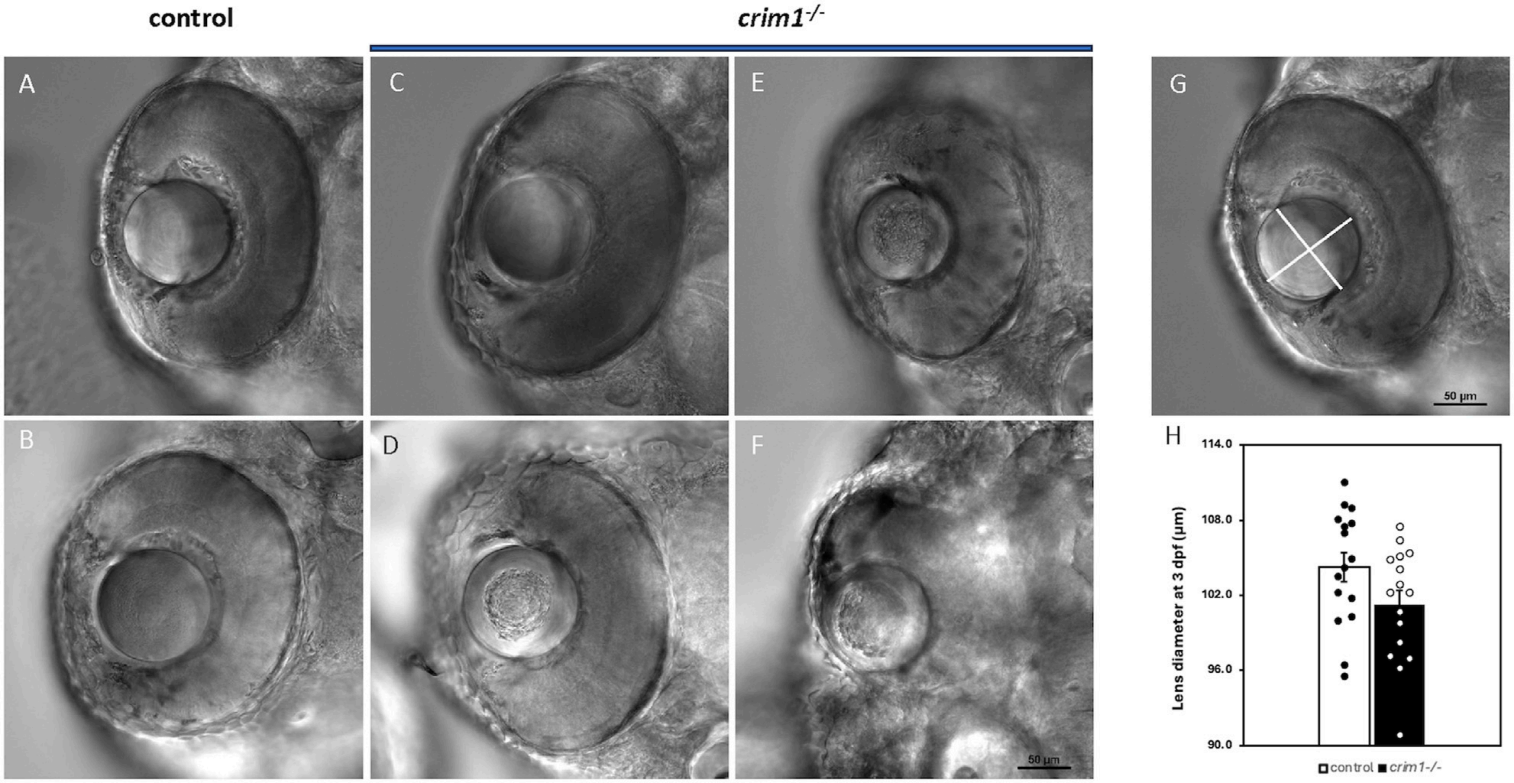
DIC microscopy of control and *crim1* ^−/−^ larvae that are homozygous for a 2 base pair deletion, c.339_340delCT p.Leu112Leufs*3, at 3 days post fertilization (dpf). **(A–C)** Lenses from control larvae and a *crim1* ^−/−^ larva that have a smooth and regular appearance. **(D–F)** Lenses from *crim1* ^−/−^ larvae that have a roughened and irregular appearance. **(G)** Method for obtaining measurements of lens diameter. **(H)** Measurements of lens diameter (in μm) in control and *crim1* ^−/−^ larvae in 16 lenses. There is a significant reduction in lens diameter for the *crim1* ^−/−^ larvae compared to controls (*P* = 0.036).

We used cryosections with H&E staining and light microscopy to examine eye and lens morphology in the *crim1* ^−/−^ larvae compared to controls at 3, 4, and 5 dpf (Figure 3). We also used differential interference contrast (DIC) microscopy to examine lens morphology in the *crim1* ^−/−^ mutant larvae compared to controls at 3 dpf (Figure 4). The lenses from *crim1* ^−/−^ mutant larvae appeared smaller in size compared to controls (Figure 3, panels A and E), and this difference in eye size persisted at 4 dpf and 5 dpf (Figure 3, panels B, C, and D and F, G, and H). The lens morphology also appeared to be irregular in *crim1* ^−/−^ mutant larvae compared with the typical spherical shape of control lenses (Figure 3, panel F compared to panel B and panel H compared to panel D). Examining the lenses with DIC microscopy showed that controls had a regular appearance of the lens in 8/8 larvae (Figure 4, panels A and B) compared to 3/14 *crim1* ^−/−^ mutant larvae that had an irregular, roughened appearance of the lens (Figure 4, panels D-F). However, some of the *crim1* ^−/−^ mutant larvae retained a normal appearance of the lens (Figure 4, panel C). The quantification of lens diameter for 16 lenses in controls and *crim1* ^−/−^ mutant larvae showed that *crim1* ^−/−^ larvae had significantly smaller lenses than controls (Figure 4, panel G, showing the method of measurement, and panel H, showing lens diameters; *P*-value = 0.036).

**FIGURE 5:**
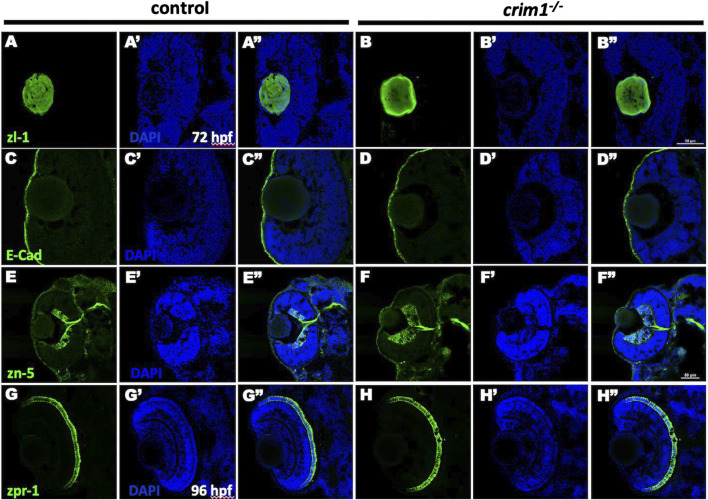
Immunohistochemistry in control and *crim1* ^−/−^ larvae that are homozygous for a 2 base pair deletion, c.339_340delCT p.Leu112Leufs*3, at 72 and 96 h post fertilization (hpf). Representative sections of eyes from control larvae **(A, C, E, G)** and *crim1* ^−/−^ larvae **(B, D, F, H)** at 72 hpf stained with zl-1 **(A, B)**, E-cadherin **(C, D)**, zn-5 antibody **(E, F)** and at 96 hpf stained with zpr-1 **(G, H)**. The lens fiber cells in the *crim1* ^−/−^ larva stained with z-l1 are less homogenous compared to the lens from a control larva **(A, B)**. The corneal epithelium showed mild clumping of cells in the *crim1* ^−/−^ larva stained with E-cadherin **(C, D)** compared to a control larva. The retinal ganglion cell layer was thicker in the *crim1* ^−/−^ larva stained with the zn-5 antibody than in a control larva **(E, F)**. There was no obvious difference in the photoreceptors in *crim1* ^−/−^ and control larvae stained with zpr-1 **(G, H)**. **(A–H)** Larvae stained with Alexa Fluor 488 dye (green). **(A′–H′)** Larvae stained with DAPI (blue). **(A′′–H′′)** Merged images. Scale bars = 50 µm.

We then examined various ocular cell types for differences between the *crim1* ^−/−^ mutant larvae and controls. Staining with the zl-1 antibody that binds to differentiating LF cells (Greiling et al., 2010) showed irregular and amorphous-appearing lenses in the *crim1* ^−/−^ larvae compared to controls (Figure 5, panels A and B). Staining of the corneal epithelium with the E-cadherin antibody that binds to the corneal epithelium was similar between controls and *crim1* ^−/−^ larvae (Figure 5, panels C and D). Retinal lamination was grossly normal when examined using the zn-5 antibody that binds to amacrine cells and retinal ganglion cells in the retinal ganglion cell layer (Figure 5, panels E and F), but there was a significant increase in thickness of the retinal ganglion cell layer in the *crim1* ^−/−^ larvae compared to controls (Supplementary Figure S2; *P*-value = 0.00034). There was no obvious difference between controls and *crim1* ^−/−^ mutant larvae using the zpr-1 antibody that binds to photoreceptor cells (Figure 4, panels G and H). We did not observe colobomas in the *crim1* ^−/−^ larvae.

**FIGURE 6:**
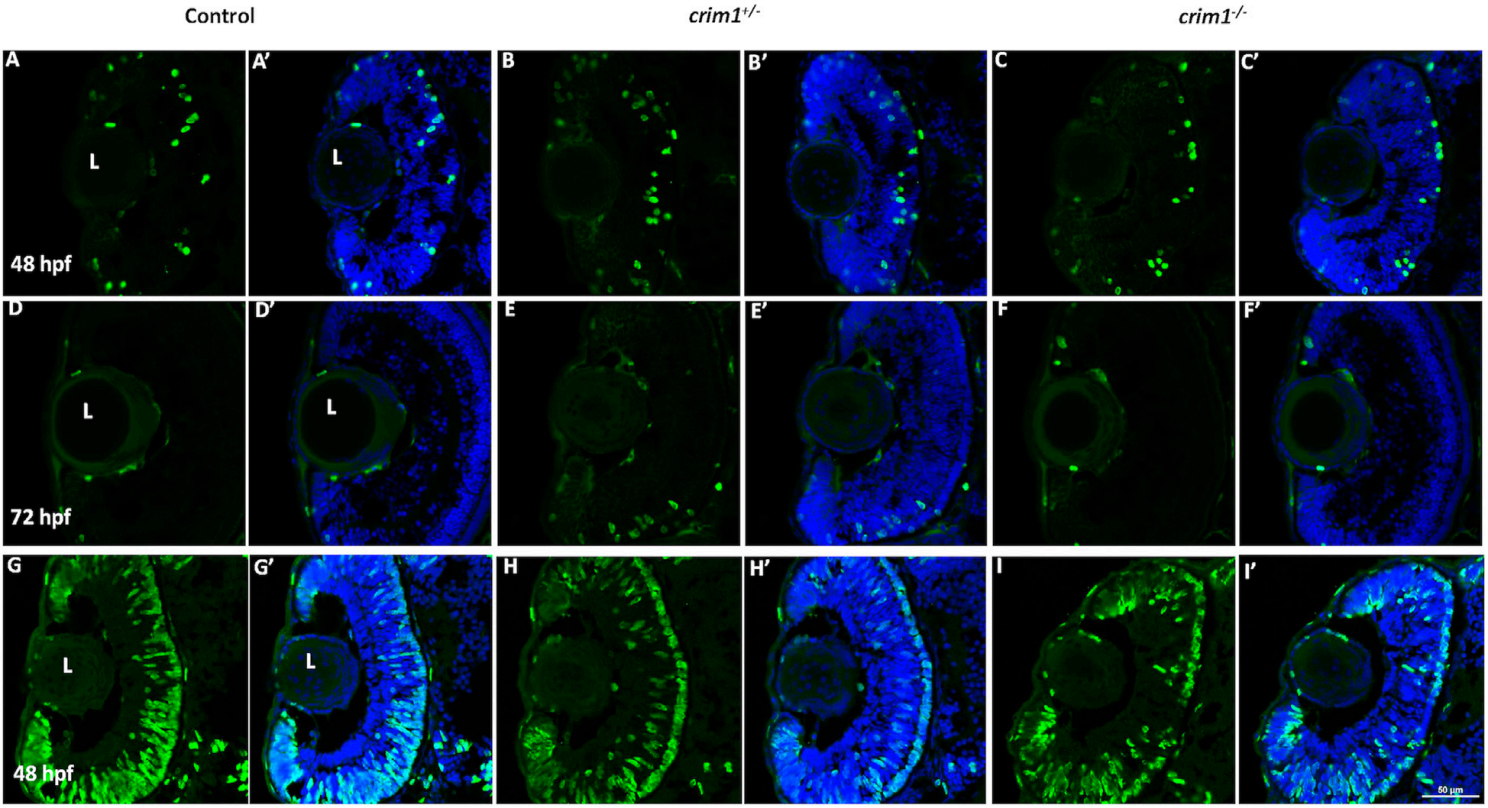
Phospho-histone H3 and 5-bromo-2′-deoxyuridine staining in control, *crim1* ^+/−^ larvae, and *crim1* ^−/−^ larvae that are homozygous for a 2 base pair deletion, c.339_340delCT p.Leu112Leufs*3, at 48 and 72 h post fertilization (hpf). Representative sections of eyes from control larvae **(A, D, G)**, *crim*1^+/−^ larvae **(B, E, H)**, and *crim1* ^−/−^ larvae **(C, F, I)** stained with the PH3 antibody (Alexa Fluor 488 dye; green) and DAPI (merged **(A’–F’)**, blue) at 48 and 72 hpf **(A–F)** and with BrdU (Alexa Fluor 488 dye; green) and DAPI (merged **(G’–I’)**, blue) at 48 hpf **(G–I)**. The quantification of the BrdU antibody showed a significant reduction in BrdU staining in the homozygous *crim1* ^−/−^ larvae compared to control at 48 hpf (*P* = 0.013). L = lens.

**FIGURE 7:**
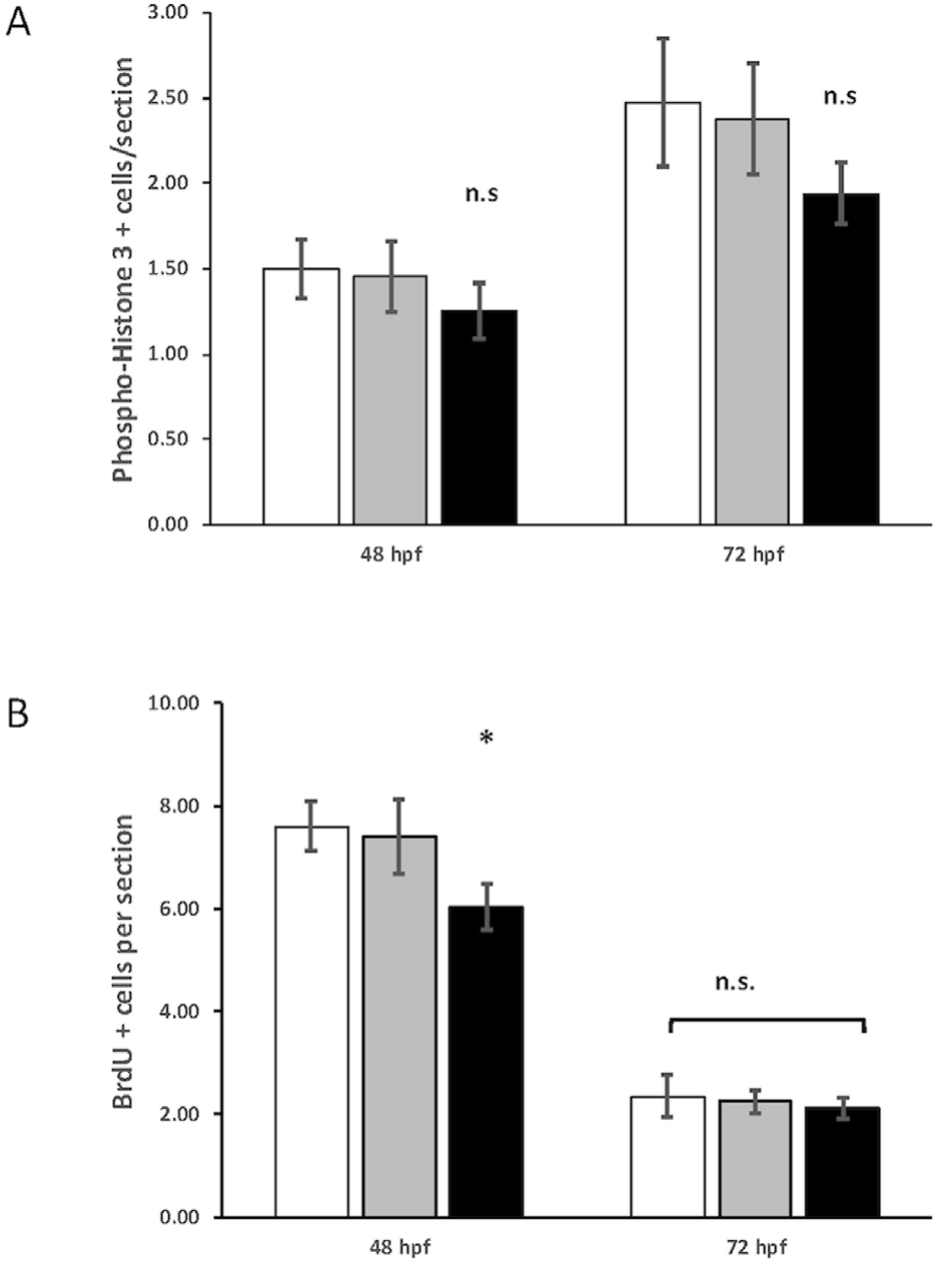
Quantification of phospho-histone H3 and 5-bromo-2′-deoxyuridine (BrdU) staining in control, heterozygous *crim1* ^+/−^, and *crim1* ^−/−^ larvae that are homozygous for a 2 base pair deletion, c.339_340delCT p.Leu112Leufs*3, at 48 and 72 h post fertilization (hpf). **(A)** The quantification of the PH3 antibody showed no significant difference between control, heterozygous *crim1* ^+/−^, and *crim1* ^−/−^ larvae. A total of 14 control larvae were scored, 11 crim1^+/−^ larvae were scored, and 8 *crim1* ^−/−^ larvae were scored at 48 hpf. A total of 19 control larvae, 8 crim1^+/−^ larvae, and 17 *crim1* ^−/−^ larvae were scored at 72 hpf. **(B)** The quantification of the BrdU antibody showed a significant reduction in BrdU staining in the homozygous *crim1* ^−/−^ larvae compared to control and heterozygous *crim1* ^+/−^ larvae at 48 hpf (*P* = 0.013) but not at 72 hpf. A total of eight control larvae were scored, three crim1^+/−^ larvae were scored, and seven *crim1* ^−/−^ larvae were scored at 48 hpf. A total of five control larvae, five crim1^+/−^ larvae, and five *crim1* ^−/−^ larvae were scored at 72 hpf.

We reasoned that the smaller eye size in the *crim1* ^−/−^ mutant larvae could be due to reduced cell proliferation. Staining with PH3 did not show any difference between *crim1* ^+/−^ heterozygous larvae and controls and between *crim1* ^−/−^ mutant larvae compared and controls (Figure 6, panels A–F; Figure 7A). There was a significant reduction in staining in *crim1* ^−/−^ mutant larvae compared to controls at 48 hpf (*P* = 0.013; Figure 6 panels G–I; Figure 7B), consistent with reduced cellular proliferation, but this difference was not statistically significant for heterozygous *crim* ^*+*/−^ larvae compared to controls at 48 hpf or between *crim1* ^−/−^ larvae compared to controls at 72 hpf.

### Bulk RNA-seq comparing gene expression between *crim1* ^−/−^ larvae and controls shows the downregulation of *clic4* and the upregulation of *fgf1b*

**FIGURE 8:**
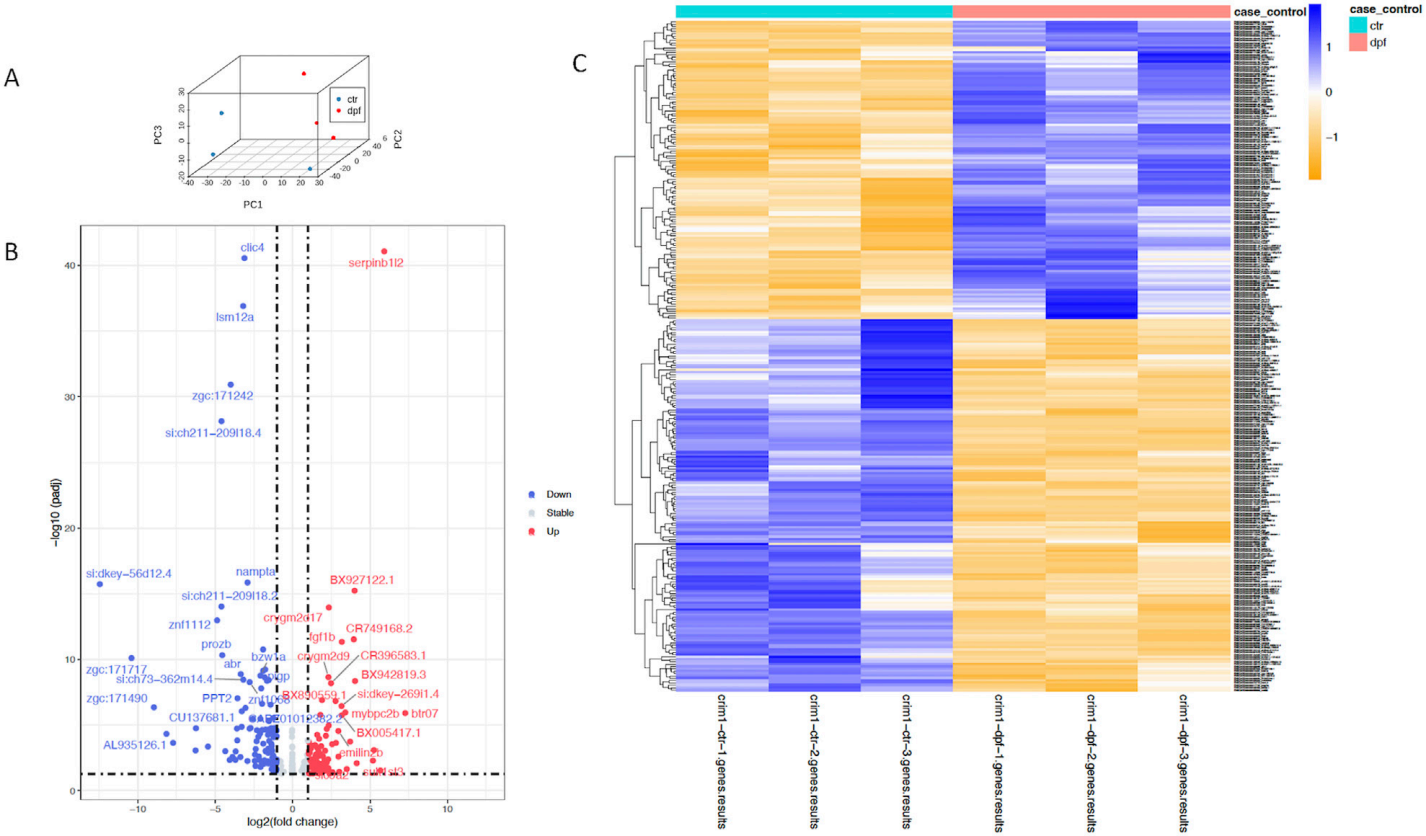
Principal component analysis, heatmap, and volcano plot showing DEGs for controls and *crim1* ^−/−^ larvae. Data from DEGs with adjusted *P*-values </ = 0.05 and an absolute (log2foldchange) >/ = 1 were used to generate the plots. **(A)** Three-dimensional PCA plot showing the separation of samples from control larval eyes (blue dots) compared to samples from *crim1* ^−/−^ larval eyes (red dots). **(B)** Volcano plot showing differential gene expression for *crim1* ^−/−^ mutant larvae and controls. The log2 fold change indicates the mean expression level for each gene. Each dot represents one gene. Selected genes with an adjusted *P*-value </ = 0.01 and absolute (log2foldchange) >/ = 2 have been named. Downregulated genes are shown in blue and upregulated genes are shown in red. **(C)** Heatmap of expressed genes in *crim1* ^−/−^ larvae and controls, showing separation of gene expression between control and *crim1* ^−/−^ mutant larvae. The type of sample (control or *crim1* ^−/−^ mutant) is listed on the X axis and the Y axis contains DEGs.

**FIGURE 9:**
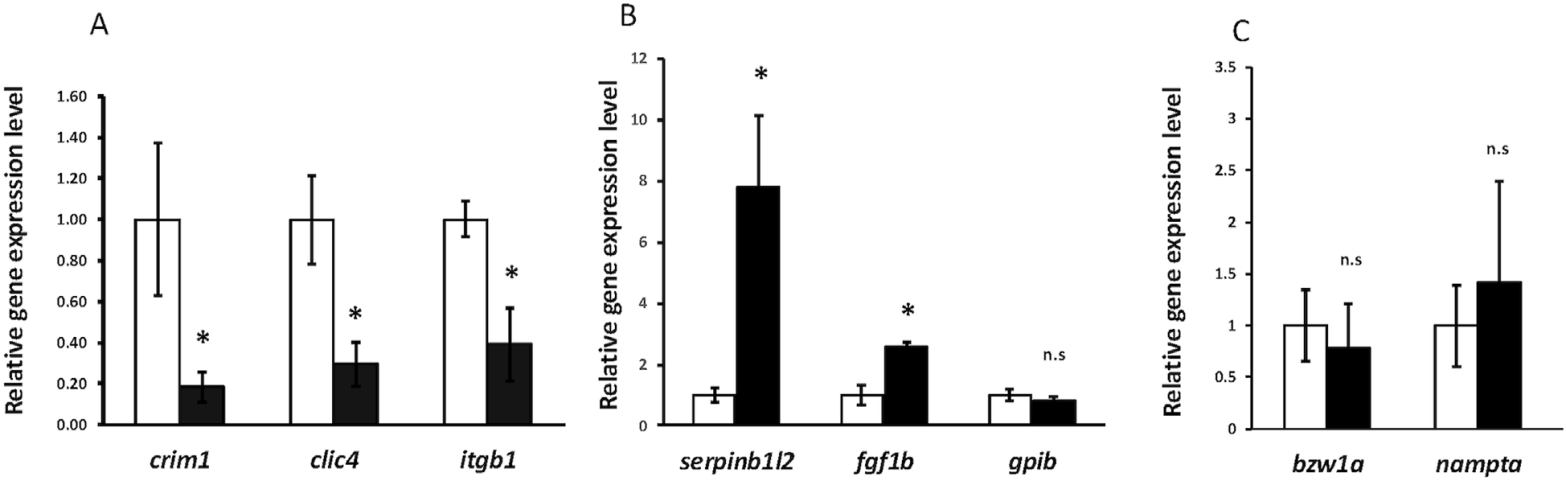
RT-qPCR showing significant downregulation of *crim1*, *clic4*, and *itgb1* and significant upregulation of *serpinb1l2* and *fgf1b*. **(A)** RT-qPCR showing expression of *crim1*, *clic4*, and *itgb1* in *crim1* ^−/−^ larvae compared to controls. RT-qPCR was performed on dissected eyes for all experiments from control (white columns; expression normalized to 1.0) and *crim1* ^−/−^ larvae (black columns) at 72 hpf. The results show significantly reduced expression of *crim1* (*P* = 0.04), *clic4* (*P* = 0.02), and *itgb1* (*P* = 0.02) in *crim1* ^−/−^ larvae compared to controls. **(B)** RT-qPCR showing expression of *serpinb1l2*, *fgf1b*, and *gpib* in *crim1* ^−/−^ larvae compared to controls. The results show significantly increased expression of *serpinb1l2* (*P* = 0.02) and *fgf1b* (*P* = 0.02) but not *gpib* (*P* = 0.25) in *crim1* ^−/−^ larvae compared to controls. **(C)** RT-qPCR showing expression of *bzw1a* and *nampta* in *crim1* ^−/−^ larvae compared to controls. The results do not show significantly reduced expression for *bzw1a* (*P* = 0.4) or *nampta* (*P* = 0.16) in *crim1* ^−/−^ larvae compared to controls.

A three-dimensional PCA plot confirmed the separation of gene expression in the *crim1* ^−/−^ samples compared to the control samples (Figure 8A). There were 223 genes that were significantly dysregulated (adjusted *P*-value < 0.05), with 109 upregulated genes and 114 downregulated genes (Supplementary Table S7). A heatmap showing the separation of the DEGs in control and *crim1* ^−/−^ larvae is shown in Figure 8C, and a volcano plot showing the distribution of dysregulated genes is shown in Figure 8B. The top 16 downregulated and upregulated DEGs and transcripts from the bulk RNA-seq experiments comparing gene expression from *crim1* ^−/−^ larval eyes with those of controls at 72 hpf are shown in Table 2. *crim1* expression was downregulated in the *crim1* ^−/−^ larvae compared to controls with an adjusted *P*-value of 1.72e^−11^ (Table 2), consistent with the predicted decrease in *crim1* expression after CRISPR targeting of this gene. This result was confirmed with RT-qPCR at 24, 48, and 72 hpf (see Figure 9A; *P*-value = 0.04; Supplementary Figure S3). Chloride intracellular channel 4 (*clic4*) was the most significantly downregulated gene, with an adjusted *P-*value of 2.77e^−41^ (Table 2). RT-qPCR also confirmed reduced expression for this gene in RNA from *crim1* ^*−/−*^ mutant eyes compared to controls (*P*-value = 0.02; Figure 9A). IHC revealed mildly reduced expression of *clic4* in the lens at the same timepoint (Supplementary Figure S4), but expression of this gene was still detectable in the *crim1* ^*−/−*^ mutant lenses.

**TABLE 2:** ‘Bulk’ RNA-seq data comparing gene expression in pooled eyes from *crim1* ^−/−^ larvae and controls at 72 h post fertilization.

Transcript	baseMean	log2fold change	lfcSE	p-value	p-value-adjusted
A. Top 16 downregulated genes and transcripts^a^					
**ENSDARG00000022995_*clic4***	**1417.65113478425**	**−3.116924394**	**0.22401195**	**2.47884473507317E-45**	**2.76862168460322E-41**
ENSDARG00000028848_*lsm12a*	942.0604358	−3.196371058	0.240794583	1.67012581206274E-41	1.24357567966191E-37
ENSDARG00000078551_zgc:171242	239.3640128	−4.001484433	0.329214951	2.15271715092427E-35	1.20218489293366E-31
ENSDARG00000090847_si:ch211-209l18.4	268.5598257	−4.598188862	0.397143293	1.64424564376678E-32	7.34583183809247E-29
ENSDARG00000030598_*nampta*	223.3367736	−2.916142534	0.32901841	3.77421115760471E-20	1.40513881397623E-16
ENSDARG00000070845_si:dkey-56d12.4	202.4324482	−12.45790992	3.210559617	5.84935470704997E-20	1.86661264922975E-16
ENSDARG00000093111_si:ch211-209l18.2	110.5653762	−4.601959188	0.554522667	3.80087976204318E-18	9.43378356939118E-15
ENSDARG00000095997_*znf1112*	66.66494953	−4.881771601	0.602438657	5.24178351878173E-17	1.06446327493224E-13
**ENSDARG00000029668_*crim1***	**371.8757595**	**−1.909419688**	**0.260435771**	**1.08105349842699E-14**	**1.72489807484729E-11**
ENSDARG00000076900_*prozb*	77.71168737	−4.548056006	0.628109279	3.14056352005347E-14	4.67692719406363E-11
ENSDARG00000087583_zgc:171717	59.32954371	−10.4138606	3.002084963	5.56844244593351E-14	7.77424170982892E-11
ENSDARG00000026985_*lims1*	871.6392918	−1.773288308	0.260568759	4.37717879264356E-13	5.75161293353363E-10
ENSDARG00000040738_zgc:153846	3749.06665230627	−1.90864069	0.282126596	5.86616373490636E-13	7.2799091950188E-10
ENSDARG00000095180_*abr*	141.0912979	−3.353128783	0.499914262	1.10568159925171E-12	1.29993239810972E-09
**ENSDARG00000010481_*bzw1a***	**10162.4901634404**	**−2.085522843**	**0.314863898**	**1.52435293004457E-12**	**1.70254978756678E-09**
ENSDARG00000061301_*gmpr2*	240.9486671	−1.806472512	0.274731382	2.23683527452586E-12	2.27120119828903E-09

B. Top 16 upregulated genes and transcripts^a^					
ENSDARG00000070396_*serpinb1l2*	248.9753889	5.917631088	0.419678053	3.79272259658149E-46	8.47218373624374E-42
ENSDARG00000100571_BX927122.1	118.3555502	4.002262291	0.460617552	2.09645534201016E-19	5.85382742872786E-16
ENSDARG00000069817_*crygm2d17*	357209.663845749	2.339474986	0.281760061	5.01199623143333E-18	1.11957971817758E-14
ENSDARG00000099160_CR749168.2	73.83098531	3.950013197	0.520080698	1.59697314618097E-15	2.97276551161587E-12
**ENSDARG00000042811_*fgf1b***	**104.0994529**	**3.178050805**	**0.421389505**	**2.63462345132932E-15**	**4.52709374275341E-12**
ENSDARG00000115701_*crygm2d9*	98024.8684670509	2.311361805	0.347528566	2.08552486980581E-12	2.21840259722486E-09
ENSDARG00000096105_BX942819.3	66.97799643	4.031620481	0.624517608	5.09323159374713E-12	4.37586951312013E-09
ENSDARG00000093732_CR396583.1	105.9471638	2.479404822	0.388995712	8.0668539212446E-12	6.43562081759864E-09
ENSDARG00000103826_*gpib*	176.4299231	1.896668576	0.321874576	1.71301346667121E-10	1.23436434898392E-07
ENSDARG00000093309_BX890559.1	66.08312647	2.771876072	0.471881391	2.08323292387602E-10	1.4542267829232E-07
ENSDARG00000100833_si:dkey-269i1.4	129.8397323	3.158241167	0.557575683	5.52012373920866E-10	3.52310068818409E-07
ENSDARG00000021265_*mybpc2b*	153.2540861	3.401163098	0.618412655	1.8877928261176E-09	1.08126964486705E-06
ENSDARG00000105558_*btr07*	40.99360149	7.274025906	1.377105293	2.17530516611294E-09	1.21479917001577E-06
ENSDARG00000071032_*pnkd*	855.3410608861	1.79015604	0.331948109	3.03357966907871E-09	1.65278299141171E-06
ENSDARG00000094215_BX005417.1	131.6544772	3.175146465	0.589768819	3.30050263976578E-09	1.75539590397828E-06
ENSDARG00000093873_BX324213.1	84.71644171	2.328856004	0.463071923	2.23813435192329E-08	1.06373287560133E-05

^a^ Genes that are discussed in the text are highlighted in bold.

Crim1 recruits and co-localizes with β1-integrin at the plasma membrane and is involved in integrin recycling and β1-integrin-dependent cell adhesion (Argenzio et al., 2014). We, therefore, performed RT-qPCR to measure the expression of *itgb1* encoding β1-integrin in the *crim1* ^−/−^ mutant eyes compared to controls. We observed significant downregulation of *itgb1* (*P*-value = 0.02; Figure 9A). We considered a model in which the reduced *clic4* expression observed in the *crim1* ^*−/−*^ larvae disrupts integrin-mediated cell interactions and investigated whether Crim1 can directly bind to Clic4. Immunoprecipitation showed a faint band at the expected size (29 kDa) in a lysate obtained from whole larval heads from controls at 72 hpf incubated with the recombinant Crim1 antibody and using the anti-Clic4 antibody for Western blotting (Supplementary Figure S5). However, the polyclonal nature of the Clic4 antibody resulted in non-specific bands, and our results do not demonstrate a definite interaction. We could not identify a suitable antibody for itgb1 to verify interactions between itgb1 and Clic4 or Crim1 (data not shown). There are multiple *itgb1* homologs in zebrafish including integrin beta 1a (itgb1a), integrin beta 1b (itgb1b), integrin beta 1b.1 (itgb1b.1), and integrin beta 1b.2 (*itgb1b.2*; Mould et al., 2006; Wang et al., 2014). The *Danio rerio* genes *itgb1a* and *itgb1b* are closely homologous to integrin beta1 subunits present in other vertebrates, but *itgb1b.1* and *itgb1b.2* are unconventional beta1 subunits that have not yet been described in other vertebrate species (Wang et al., 2014).

**FIGURE 10:**
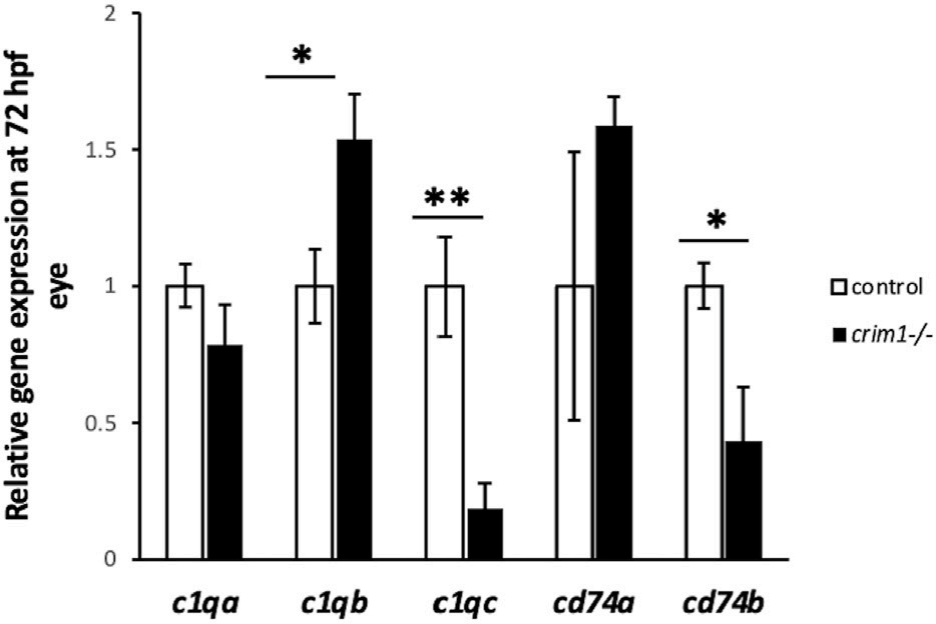
*c1q* levels measured by RT-qPCR in control and *crim1* ^−/−^ larvae that are homozygous for a 2 base pair deletion, c.339_340delCT p.Leu112Leufs*3, at 72 h post fertilization (hpf). RT-qPCR showing expression of *c1qa*, *c1qb*, *c1qc*, *cd74a*, and *cd74b* in *crim1* ^−/−^ larvae compared to controls. RT-qPCR was performed on dissected eyes for all experiments from control (white columns; expression normalized to 1.0) and *crim1* ^−/−^ larvae (black columns) at 72 hpf. The results show significantly increased expression of *c1qb* (*P* = 0.018) and significantly reduced expression of *c1qc* (*P* = 0.003) and *cd74b* (*P* = 0.032) in *crim1* ^−/−^ larvae compared to controls. *c1qa* (*P* = 0.154) and *cd74a* (*P* = 0.391) expression was not significantly different.

Our bulk RNA-seq results also showed significant upregulation of *fgf1b*, with an adjusted *P*-value of 4.53e^−12^ (Table 2). The upregulation of this gene was confirmed by RT-qPCR (*P*-value = 0.023; Figure 9B). IHC using an antibody for Fgf1 supported the increased expression of *fgf1b* in the *crim1* ^−/−^ mutant lenses, with the distribution of signal throughout the lens rather than confined to the exterior of the lens (Supplementary Figure S6). However, it is also possible that this antibody would also detect *fgf1a*. Additional upregulated genes included *serpinb1l2*, with an adjusted *P*-value of 8.47e^−42^ (Table 1B). RT-qPCR confirmed significantly increased expression for *serpinb1l2* (*P*-value = 0.02) in the *crim1* ^−/−^ mutant eyes compared to controls but did not confirm significance for the other upregulated genes, such as *gpib*, *bzw1a*, and *nampta* (Figures 9B, C). We also examined *c1q* expression in our bulk RNA-seq data in light of results demonstrating the dysregulation of complement genes in HZ mice with a hypomorphic *Crim1* allele compared to heterozygotes (*Crim1* ^glcr11^; GEO GSE62561 dataset; Zhang et al., 2023). *c1q* was significantly upregulated in our bulk RNA-seq data (*P* = 0.03; Supplementary Table S7). We, therefore, determined to verify this RNA-seq result using RT-qPCR (Figure 10) and an ELISA (Supplementary Figure S7). The RT-qPCR showed significant upregulation of *c1qb* (Figure 10; *P* = 0.018) and downregulation of *c1qc* (*P* = 0.003) and *cd74b* (*P* = 0.032). The expression of *c1qa* (*P* = 0.154) and *cd74a* (*P* = 0.391) was not significantly different. The ELISA results did not demonstrate significant dysregulation of the C1q protein in the *crim1* ^−/−^ larvae (Supplementary Figure S7). For the Gene Ontology (GO) analysis (Supplementary Figure S8), a false discovery rate (FDR) *P*-value of <0.05 was used to determine significance. Only the category ‘molecular functions’ among the three categories of GO analysis (biological processes, cellular locations, and molecular functions) had significant results (FDR < 0.05). One of the significant results under this category was the ‘structural constituent of the eye lens’ (FDR was 1.27 E^-02^).

### *crim1* ^−/−^ larvae demonstrate increased apoptosis compared to controls

Finally, we reasoned that the reduction in eye size could be associated with an increase in apoptosis in the lenses and eyes of the *crim1* ^−/−^ larvae. Examination and quantification of apoptotic cells using cleaved caspase-3 by IHC at 24, 30, 48, and 72 hpf demonstrated a significant increase in cleaved caspase-3 cells at 24 and 72 hpf but not at 30 and 48 hpf in *crim1* ^−/−^ larvae compared to controls (Supplementary Figures S9A, B). We also performed a TUNEL assay at 48 and 72 hpf and found no significant difference between the control and *crim1* ^−/−^ larvae (data not shown).

## Discussion

We present three new patients with heterozygous deletions and truncating variants predicting haploinsufficiency for *CRIM1*. We report the first truncating variant (c.2701delC p.Leu901Cys*22) in *CRIM1* in a father and son with glaucoma and optic disc pallor (patients 2 and 3; Table 1; Supplementary Table S6), strengthening the association between these ocular anomalies and haploinsufficiency for *CRIM1*. Other ocular findings included colobomas, cataracts, microcornea, cornea plana, and retinal detachment that caused reduced visual acuity with myopia, temporal visual field loss, and an afferent pupillary defect (Table 1). Haploinsufficiency for *CRIM1* has previously been associated with MACOM syndrome (Toker et al., 2003; Beleggia et al., 2015; Haug et al., 2021), but pathogenic deletions involving this gene remain rare. When the clinical data from the three new patients in this paper are examined with the 11 patients described by Toker et al. (2003) and Beleggia et al. (2015) and with the three patients reported by Haug et al. (2021), no patient had significant growth or developmental concerns (Supplementary Table S6). These findings suggest that *CRIM1* haploinsufficiency is likely to be associated with a variable phenotype that can include both macrophthalmia and microphthalmia.

We generated *crim1* ^−/−^ mutant zebrafish larvae with reduced *crim1* function due to a 2bp deletion in exon 2 and observed small eyes with small and misshapen lenses compared to controls (Figures 3, 4). In addition, we observed shortening of the bodies and increased body curvature in the *crim1* ^−/−^ larvae; in particular, body length appeared to be more affected than eye size (Figure 2D). These findings are consistent with previously reported MO models of reduced *crim1* function (Supplementary Table S2; Kinna et al., 2006; Brastrom et al., 2019). The ratio of eye size to head size was also increased in the *crim1* ^−/−^ larvae at 2 dpf, which may resemble the macrophthalmia described in MACOM (Figure 2E). Our model has strengths as we successfully bred HZ adult fish, but not all *crim1* ^−/−^ larvae were affected, and the ocular phenotype was not fully penetrant (for example, Figures 1A, 4C). Although haploinsufficiency is sufficient to cause a phenotype in patients, we did not observe eye or body malformations in heterozygous *crim1* ^+/−^ larvae, and it is possible that there are differences in genetic compensation for different species for reduced *crim1* function.

The eye findings in the *crim1* ^−/−^ larvae also resembled those observed in mouse models of *Crim1* loss of function, which have been considered useful models for the structural eye defects observed in patients with *CRIM1* haploinsufficiency. However, although congenital cataracts have been observed in *Crim1* conditional and null mutant mouse models (Pennisi et al., 2007; Chiu et al., 2012; Beleggia et al., 2015; Tam et al., 2018), they have not yet been observed in patients. We observed smaller lenses with an irregular external appearance in *crim1* ^−/−^ larvae. The variation in the eye defects in the different species might reflect differences in ocular development, in modifying alleles, or in *CRIM1*/*Crim1/crim1* expression and function. We also did not model the variant detected in two of the patients. Finally, we examined the *crim1* ^−/−^ larvae at 72 hpf, and it is also possible that lens opacities will become more visible in the *crim1* ^−/−^ larvae with increasing age.

Lens growth is characterized by epithelial cell proliferation in the germinative zone, a narrow cellular region that rings the lens epithelium toward the periphery of the anterior lens surface (Remington and Meyer, 2007). The LE cells then differentiate into secondary lens fibers in the transitional zone, undergoing cellular elongation, loss of nuclei and organelles, and synthesis of lens fiber-specific proteins, such as β- and γ-crystallins (Lovicu and McAvoy, 2005). *Crim1* is critical for the maintenance of the lens epithelium, which was anteriorly restricted in murine Crim1 larvae due to early differentiation of the LE cells into LF cells (Tam et al., 2018). Murine *Crim1* ^glcr11^, *Crim1* ^null^, and *Crim1* ^cko^ mutants demonstrated reduced numbers of LE cells, with defective polarity and proliferation (Supplementary Table S1; Beleggia et al., 2015; Zhang et al., 2016; Tam et al., 2018). Crim1 function is also required for cell adhesion between LE cells and between LE and LF cells (Zhang et al., 2016). Our data also support reduced proliferation and numbers of LE cells in the smaller lens sizes of the *crim1* ^−/−^ larvae.

The results of our RNA-seq experiments showed *clic4* as achieving the greatest significance among the downregulated genes (Table 2). Human CLIC4 encodes a 253 amino acid protein that belongs to the six-member, mammalian CLIC family (CLIC1–6) of globular proteins that are involved in gene regulation and signal transduction, cell adhesion and migration, and membrane remodeling (He et al., 2011; Argenzio et al., 2014; Argenzio and Innocenti, 2020; Chuang et al., 2022; Olotu et al., 2022). CLIC4 is present in the cytosol or on intracellular organelles (Gururaja Rao et al., 2018), but in the presence of activators, it can translocate to lipid rafts in the plasma membrane where it interacts with ezrin–radixin–moesin (ERM) proteins that connect the membrane proteins with the actin cytoskeleton (Argenzio et al., 2018; Olotu et al., 2022). Crim1 localized to the sites of actin cytoskeleton remodeling at the leading edge of cell protrusions and regulated the level of β1 integrin and focal adhesion kinase (FAK) and the extracellular signal-regulated kinase (ERK) signaling in LE cells (Iyer et al., 2016; Zhang et al., 2016). The interaction between Crim1 and integrins at the lateral surfaces of LE cells provides a mechanism for cell adhesion between LE cells and may be relevant to eye defects, including coloboma. Experiments crossing postnatal day (*P)0–3.9-GFPCre;Crim1* ^flox/flox^ and *Itgb1* ^flox/flox^ mice showed that 1 out of 4 *GFPCre;Crim1* ^flox/+^ *;Itgb1* ^flox/+^ mice displayed iris coloboma and 6/6 P21 compound heterozygotes exhibited bilateral cataracts compared to none of the four littermate controls (Zhang et al., 2016). Immunostaining of the lenses in the compound heterozygous *GFPCre;Crim1* ^flox/+^ *;Itgb1* ^flox/+^ mice showed the detachment of the LE cells from the LF cells (Zhang et al., 2016). An LE cell line was also found to have high expression of *ITGB1* (Weatherbee et al., 2019) and a mouse mutant for *Itgb1* exhibited loss of LE cells (Simirskii et al., 2007). It is also notable that beta1-integrin signaling is essential for lens fiber survival (Samuelsson et al., 2007).

We hypothesized that the downregulated *clic4* expression in the *crim1* ^*−/−*^ larvae could disrupt integrin-mediated cell interactions and that Crim1, Clic4, and Itgb1 would co-localize at sites of LE/LF cell adhesion. We used IP to investigate whether Crim1 could bind directly to Clic4 but failed to demonstrate a convincing interaction. Interestingly, Clic4 is enriched in the proximal tubule epithelial cells of the kidney, and Clic4-null murine embryos have impaired renal tubulogenesis (Chou et al., 2016). It is plausible that the reduction in *clic4* observed in our model may be relevant to the renal defects observed in one of the patients.

The bulk RNA-seq data also showed significant upregulation of *fgf1b*, with an adjusted *P*-value of 4.53 × 10^−12^ (Table 2), and increased expression of this gene was also confirmed by RT-qPCR (Figure 9B; *P* = 0.022). Lens growth is known to be regulated by genes from the FGF family (Lovicu et al., 1997; Lovicu and McAvoy, 2005; McAvoy et al., 2017), and Fgf1 has been shown to promote LE cell proliferation and fiber differentiation in explant experiments (McAvoy and Chamberlain, 1989; Qu et al., 2011). In addition, transgenic mice overexpressing Fgf1 and Fgf2 in the lens exhibited abnormal lens growth and premature differentiation of the anterior LE cells (Robinson et al., 1995; Lovicu and Overbeek, 1998; Robinson et al., 1998). The upregulation of *fgf1b* could be consistent with the known role of *crim1* in the maintenance of the anterior lens epithelium, with reduced *crim1* leading to the upregulation of other genes with similar functions.

The basic leucine zipper domain- and W2 domain-containing protein 1 (*BZW1*) gene, known as *BAZP45*, *KIAA0005*, and *5MP2* (OMIM #619252), was also downregulated in the bulk RNA-Seq data (Table 2). This gene is a member of the superfamily of bZIP transcription factors and encodes a 419 amino acid protein with a basic, leucine-zipper domain located at the N-terminal end of the protein (Mitra et al., 2001). The overexpression of *BZW1* promoted ATF4 expression in human cells, a protein that is part of the unfolded protein response (UPR) activation (Kozel et al., 2016). The UPR is active in LF cell differentiation (Firtina and Duncan, 2011), and the ectopic expression of *atf4* in *Xenopus* embryos suppressed eye formation, suggesting that tightly controlled Atf4 activity is critical for early eye patterning (Liu et al., 2011). However, our RT-qPCR results did not confirm significant dysregulation of *bzw1* (Figure 9C). The most significant upregulated gene was *serpinb1l2*, with an adjusted P-value of 8.47 × 10^−42^ (Table 2), but we could not find an exact match to an orthologous zebrafish gene in the ZFIN database.

We also considered a prior study examining murine gene expression data at postnatal day (P) 60 comparing HZ mice with a hypomorphic *Crim1* allele (*Crim1* ^glcr11^; GEO GSE62561 dataset) and using heterozygous mice as controls (Zhang et al., 2023). The previous study showed 750 DEGs, comprising 407 genes with increased expression and 343 genes with reduced expression (Zhang et al., 2023). GO analysis showed that the DEGs were primarily connected with the immune system and involved activities such as antigen processing and presentation, apoptosis, and cell translation (Zhang et al., 2023). *C1qa*, *C1qb*, *C1qc*, and *Cd74* exhibited the highest degrees of altered expression (Zhang et al., 2023). Conditional knock-out mice with a *Clic4*-null allele in the retinal pigment epithelium (RPE^∆*Clic4*^ mice) develop the clinical, histological, and functional hallmarks of dry age-related macular degeneration (Chuang et al., 2022), and complement is also known to play an important role in the pathogenesis of age-related macular degeneration (Fritsche et al., 2010). Our bulk RNA-seq results did show significant upregulation of *c1q* (*P* = 0.03) in the *crim1* ^*−/−*^ mutant larvae, but, apart from *c1qb*, we could not confirm the upregulation of complement-associated genes by RT-qPCR or ELISA. The different results may be explained by the selection of different tissues, variation in experimental timing, and variation between mRNA and protein levels.

## Conclusion

We present three new patients with ocular findings, including retinal coloboma, optic disc pallor, and glaucoma, associated with gene deletions and truncating variants in *CRIM1*. The clinical features observed in these individuals support a broader ocular phenotype associated with *CRIM1* haploinsufficiency. We generated a zebrafish model of *crim1* deficiency, in which homozygous *crim1* ^*−/−*^ mutant larvae had smaller eyes and lenses compared to controls, resembling prior MO models of reduced *crim1* expression. Bulk RNA-seq showed significant downregulation of *clic4* expression that may perturb β-integrin-mediated cell adhesion, but we could not demonstrate an interaction between Crim1 and Clic4. Our work supports the association between *CRIM1* haploinsufficiency and eye defects and has developed a stable *crim1* loss-of-function model for future research.

## Data Availability

The data presented in this study are deposited to the GEO repository, accession number GSE289562.

## Supplementary material

SUPPLEMENTARY FIGURE S1 Chromatograms showing tracings from wildtype, heterozygous, and homozygous *crim1* ^−/−^ larvae with a 2 base pair deletion, c.339_340delCT p.Leu112Leu*fs**3 (NM_212821.1) in *crim1*. **(A)** Chromatogram from wild-type larva. **(B)** Chromatogram from heterozygous *crim1* ^−/+^ larva. **(C)** Chromatogram from homozygous *crim1* ^−/−^ larva.

SUPPLEMENTARY FIGURE S2 Immunohistochemistry with zn-5 in control and *crim1* ^−/−^ larvae that are homozygous for a 2 base pair deletion, c.339_340delCT p.Leu112Leu*fs**3 in *crim1*, at 72 h post fertilization (hpf). **(A, B)** Two or three measurements were taken from at least three control and *crim1* ^−/−^ larvae and quantified by measuring the width of the white lines. Supplementary Figure S2C. The results show a significant increase in the width of the retinal ganglion cell layer (stained in green) in *crim1* ^−/−^ larvae compared to controls (*P* = 0.00034).

SUPPLEMENTARY FIGURE S3 Time course of *crim1* expression in control and *crim1* ^−/−^ larvae that are homozygous for a 2 base pair deletion, c.339_340delCT p.Leu112Leu*fs**3 in *crim1*, at 24, 48, and 72 h post fertilization (hpf). RT-qPCR showing the expression of *crim1* in *crim1* ^−/−^ larvae compared to controls. RT-qPCR was performed on dissected eyes for all experiments from control (white columns; expression normalized to 1.0) and *crim1* ^−/−^ larvae (black columns) at 24, 48, and 72 hpf. The results show significantly reduced expression of *crim1* (*P* = 0.0164 at 24 hpf, *P* = 0.0077 at 48 hpf, and *P* = 0.0122 at 72 hpf) in *crim1* ^−/−^ larvae compared to controls.

SUPPLEMENTARY FIGURE S4 Immunohistochemistry with Clic4 in control and *crim1* ^−/−^ larvae that are homozygous for a 2 base pair deletion, c.339_340delCT p.Leu112Leu*fs**3 in *crim1*, at 72 h post fertilization (hpf). Representative sections of eyes from control larvae **(A, B)** and *crim1* ^−/−^ larvae **(C, D)** at 72 hpf. The first two panels **(A, C)** were negative sections without antibodies, and the second two panels **(B, D)** were stained with an antibody against Clic4. There is a mild reduction in *clic4* expression in *crim1* ^−/−^ larvae compared to control larvae, but this expression is still present in the *crim1* ^−/−^ mutant larvae.

SUPPLEMENTARY FIGURE S5 Lysate from whole larval heads at 72 hpf incubated with the recombinant anti-crim1 antibody for immunoprecipitation, followed by Western blotting using the anti-clic4 antibody. A faint band can be observed at 29 kDa, the expected size, but there are other more intense bands.

SUPPLEMENTARY FIGURE S6 Immunohistochemistry using an antibody to Fgf1 in control and *crim1* ^−/−^ larvae that are homozygous for a 2 base pair deletion, c.339_340delCT p.Leu112Leu*fs**3 at 48 h post fertilization (hpf). Representative sections of eyes from control larvae **(A, B)** and *crim1* ^−/−^ larvae **(C, D)**. There is an increase in *fgf1* staining in the smaller and irregular lenses in the *crim1* ^−/−^ larvae compared to control larvae.

SUPPLEMENTARY FIGURE S7 C1q levels measured with an ELISA in control and *crim1* ^−/−^ larvae that are homozygous for a 2 base pair deletion, c.339_340delCT p.Leu112Leu*fs**3, at 12, 24, 48, and 72 h post fertilization (hpf). Mean C1q levels, measured in µg/mL using an ELISA, in control larvae are indicated by the white columns, and mean C1q levels in the *crim1* ^−/−^ mutant larvae are indicated by the black columns. There is no significant difference (n.s.) between the C1q levels in controls and *crim1* ^−/−^ mutant larvae at any time period.

SUPPLEMENTARY FIGURE S8 GO analysis shown as a bar plot using Ensembl gene identities of DEGs. A false discovery rate *P*-value of <0.05 was used to determine significance. Only the category ‘molecular functions’ among the three categories of GO analysis (biological processes, cellular locations, and molecular functions) had significant results (FDR < 0.05). In this category, the ‘structural constituent of the eye lens’, achieved significance (FDR was 1.27E^-02^).

SUPPLEMENTARY FIGURE S9 Immunohistochemistry with cleaved caspase-3 in control and *crim1* ^−/−^ larvae that are homozygous for a 2 base pair deletion, c.339_340delCT p.Leu112Leu*fs**3 at 24 and 30 h post fertilization (hpf). **(A)** Representative sections of eyes from control larvae (**(A, B)** at 24 hpf and **(E, F)** at 30 hpf) and *crim1* ^−/−^ larvae (**(C, D)** at 24 hpf and **(G, H)** at 30 hpf). Increased staining can be observed throughout the lenses of the *crim1* ^−/−^ mutant larvae, compared to reduced staining localized to the anterior portion of the lens in controls. **(B)** Quantification of cleaved caspase-3/nuclei in control and *crim1* ^−/−^ larvae that are homozygous for a 2 base pair deletion, c.339_340delCT p.Leu112Leu*fs**3, at 24, 30, 48, and 72 h post fertilization (hpf). Control results are represented by white columns, and *crim1* ^−/−^ larvae results are represented by black columns. There is a statistically significant difference between control and *crim1* ^−/−^ larvae at 24 and 72 hpf.
