# Supplementary figures and images for: A zebrafish model of crim1 loss of function has small and misshapen lenses with dysregulated clic4 and fgf1b expression
Source: Front Cell Dev Biol. 2025 Mar 6;13:1522094. doi: 10.3389/fcell.2025.1522094 (PMC11922885; doi:10.3389/fcell.2025.1522094)

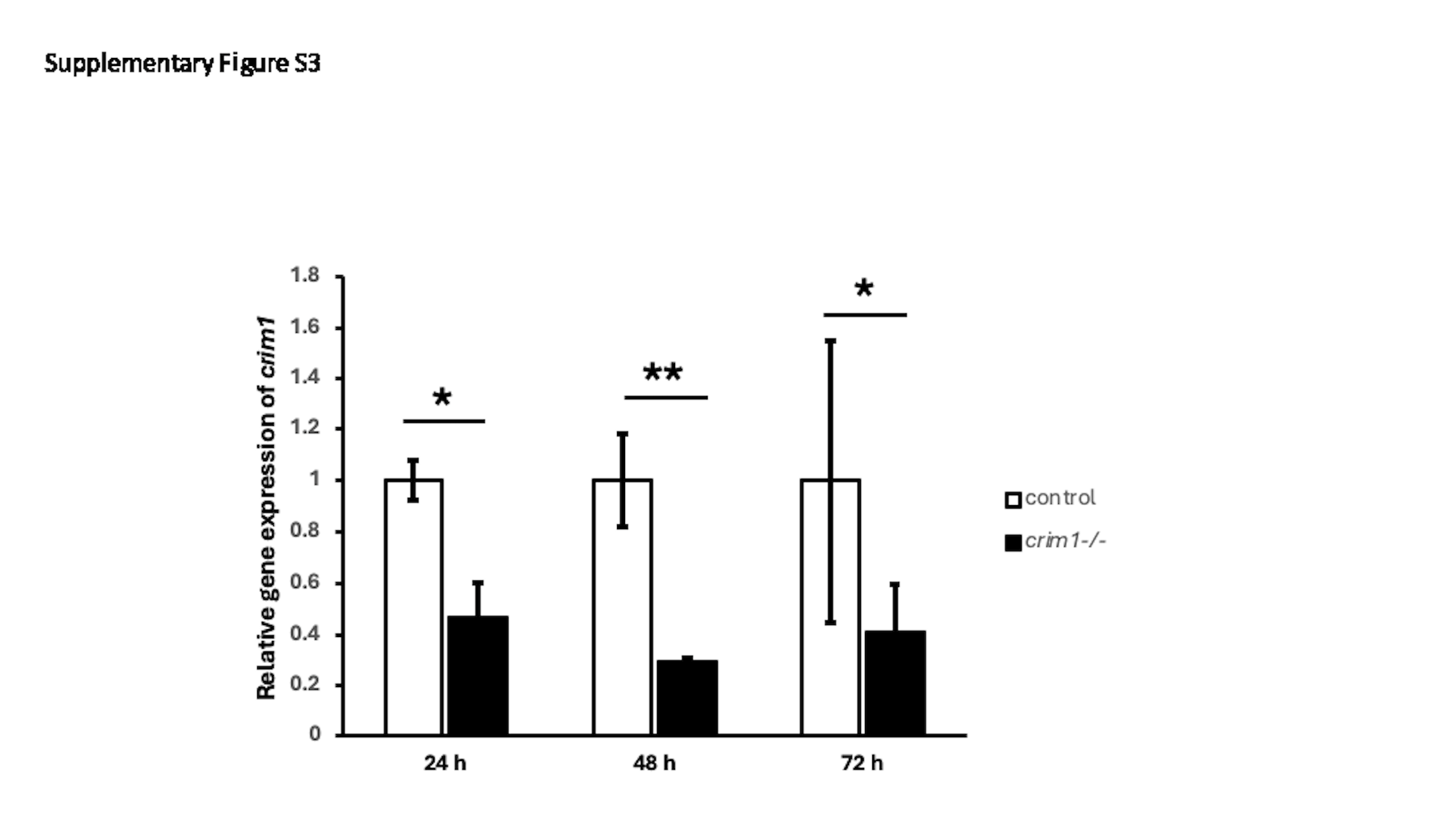

Supplement: Supplementary file 1 [file Image3.tiff]

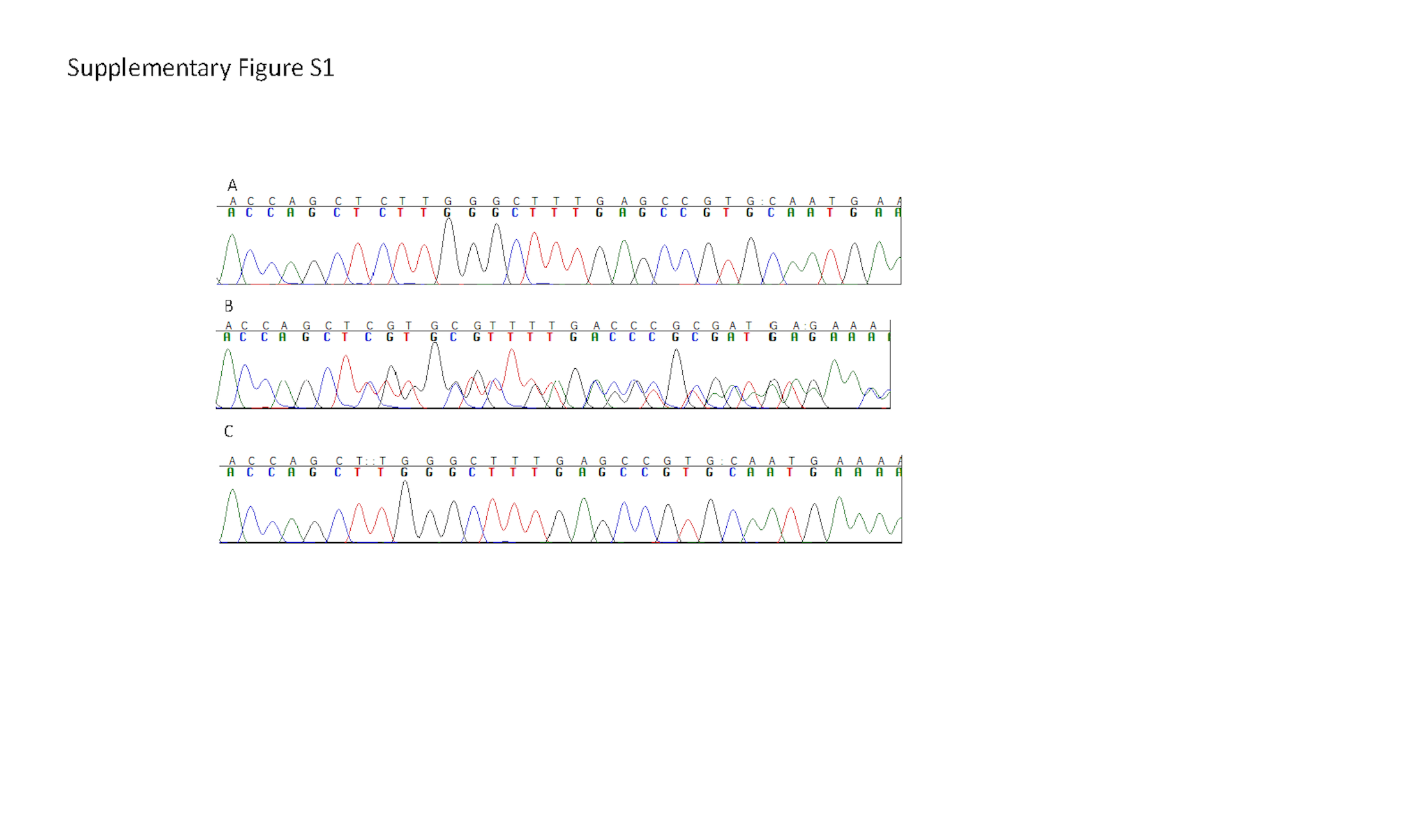

Supplement: Supplementary file 2 [file Image1.tiff]

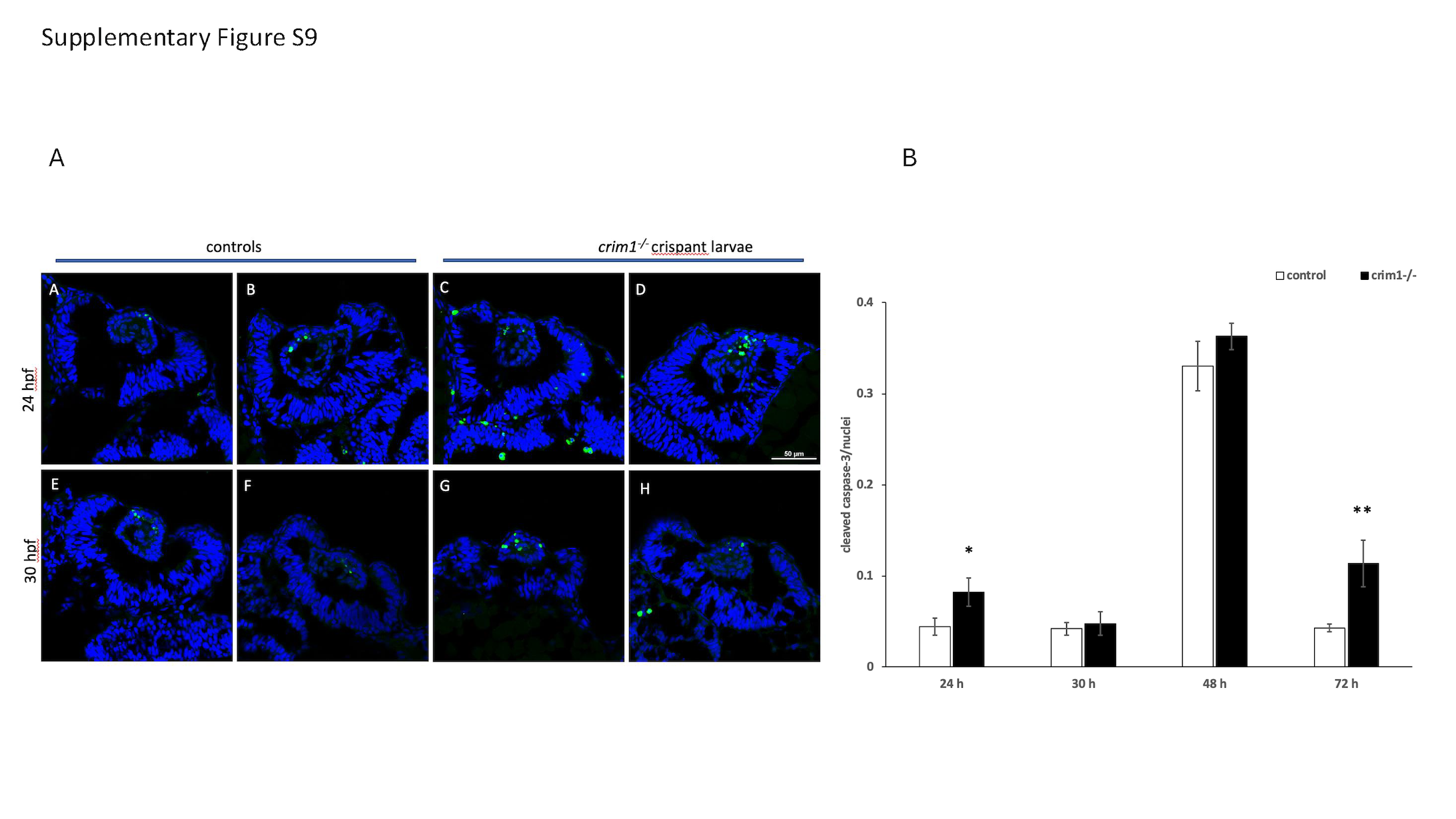

Supplement: Supplementary file 3 [file Image9.tiff]

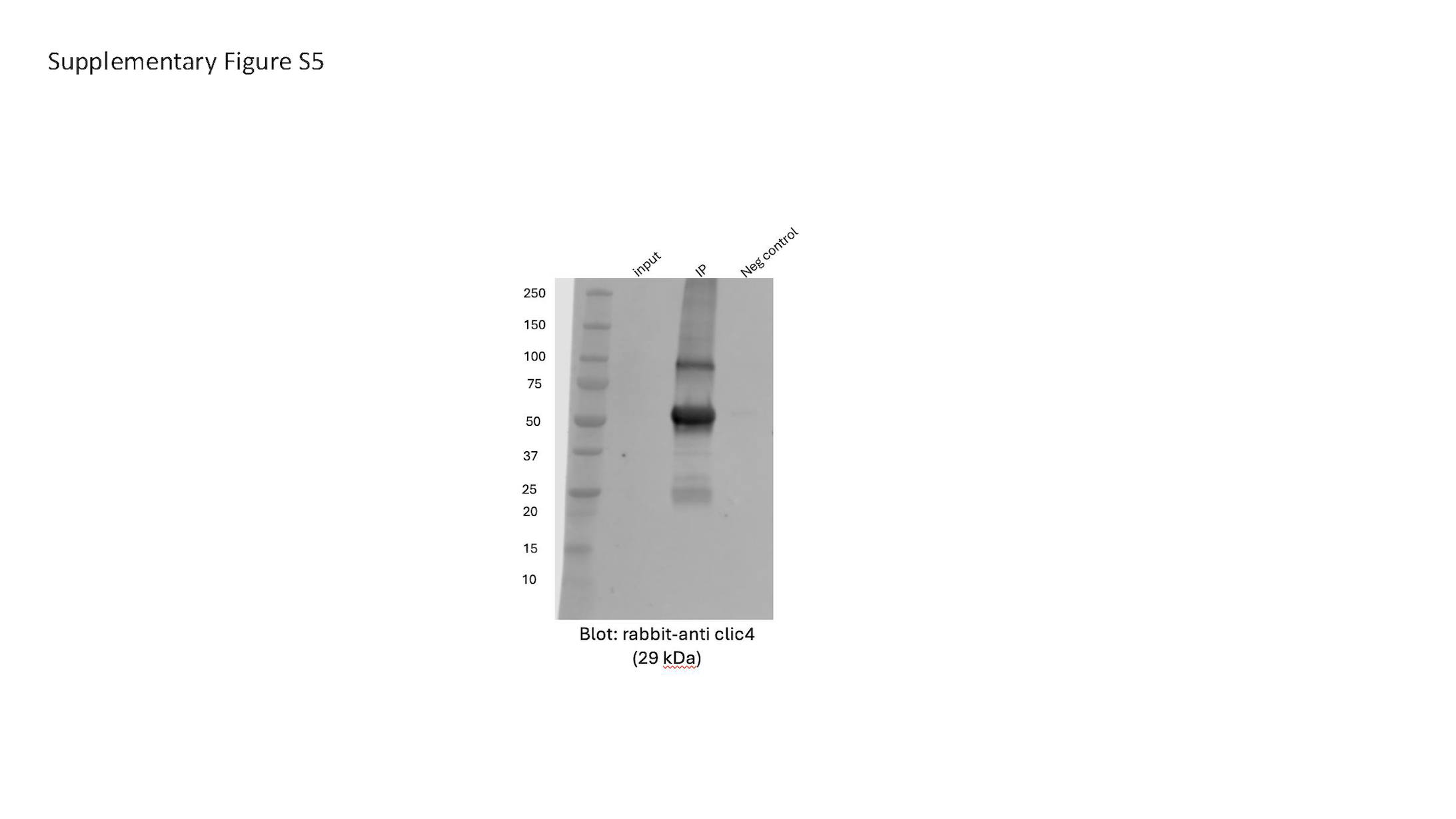

Supplement: Supplementary file 5 [file Image5.tiff]

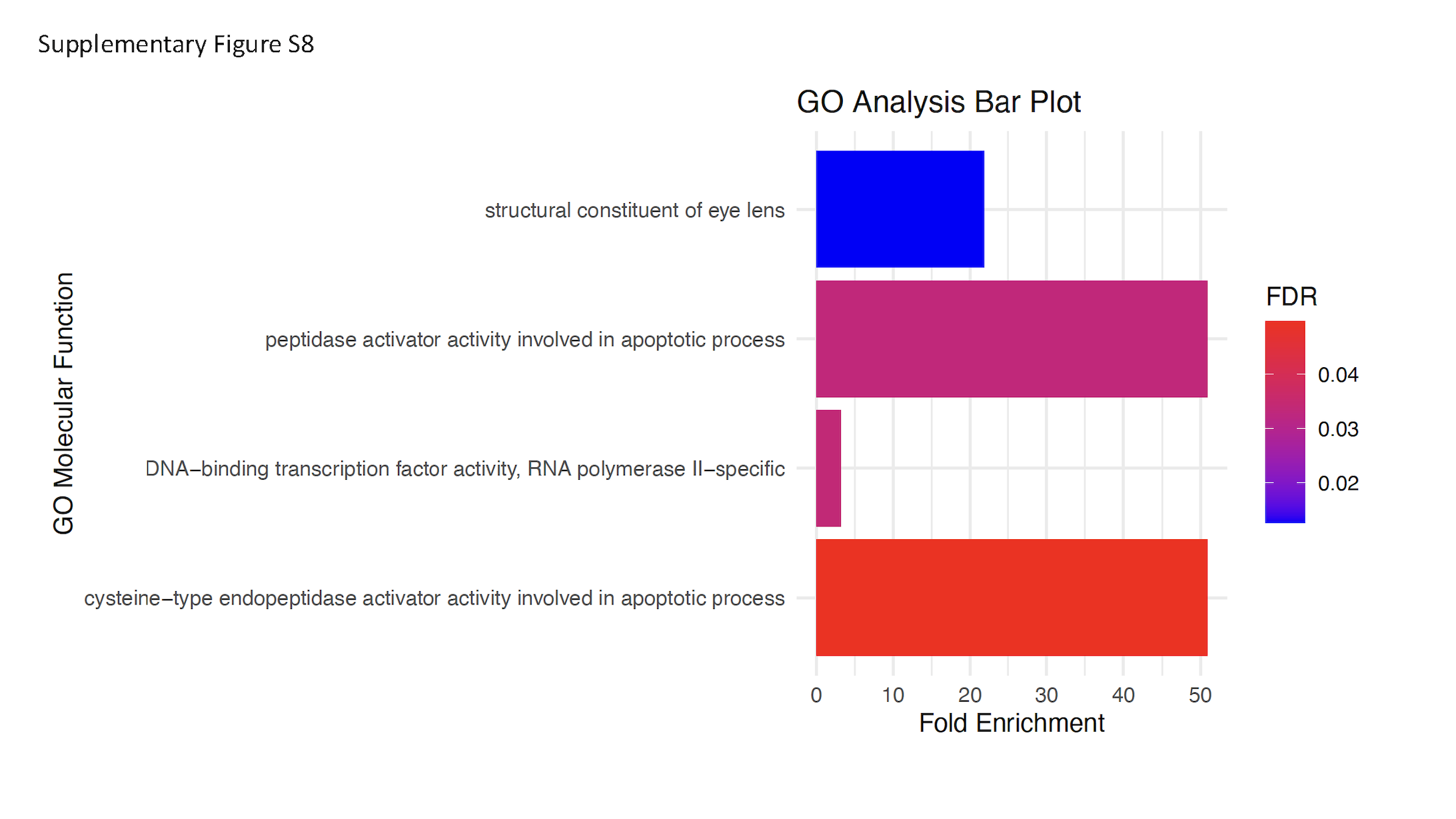

Supplement: Supplementary file 6 [file Image8.tiff]

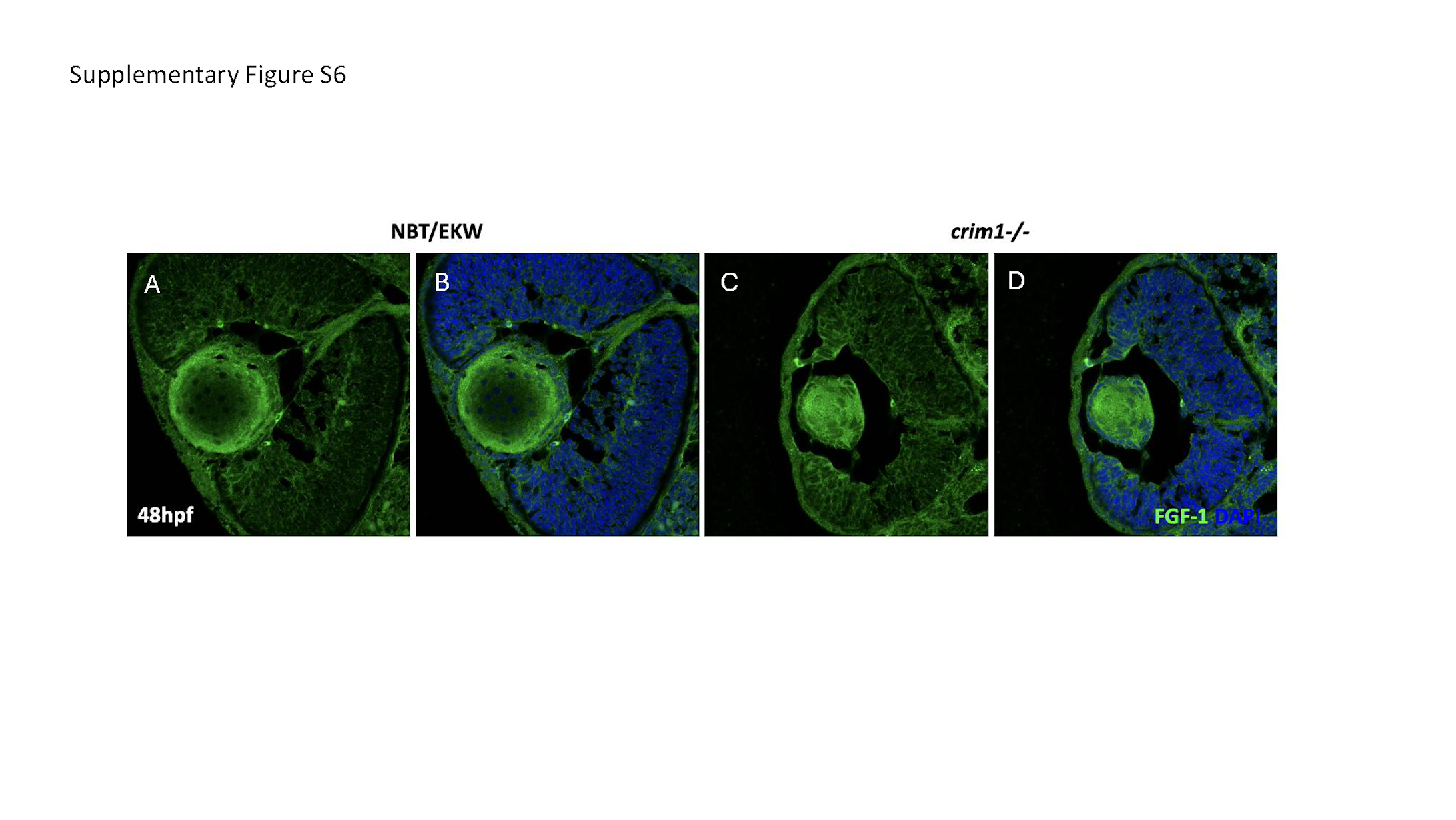

Supplement: Supplementary file 12 [file Image6.tiff]

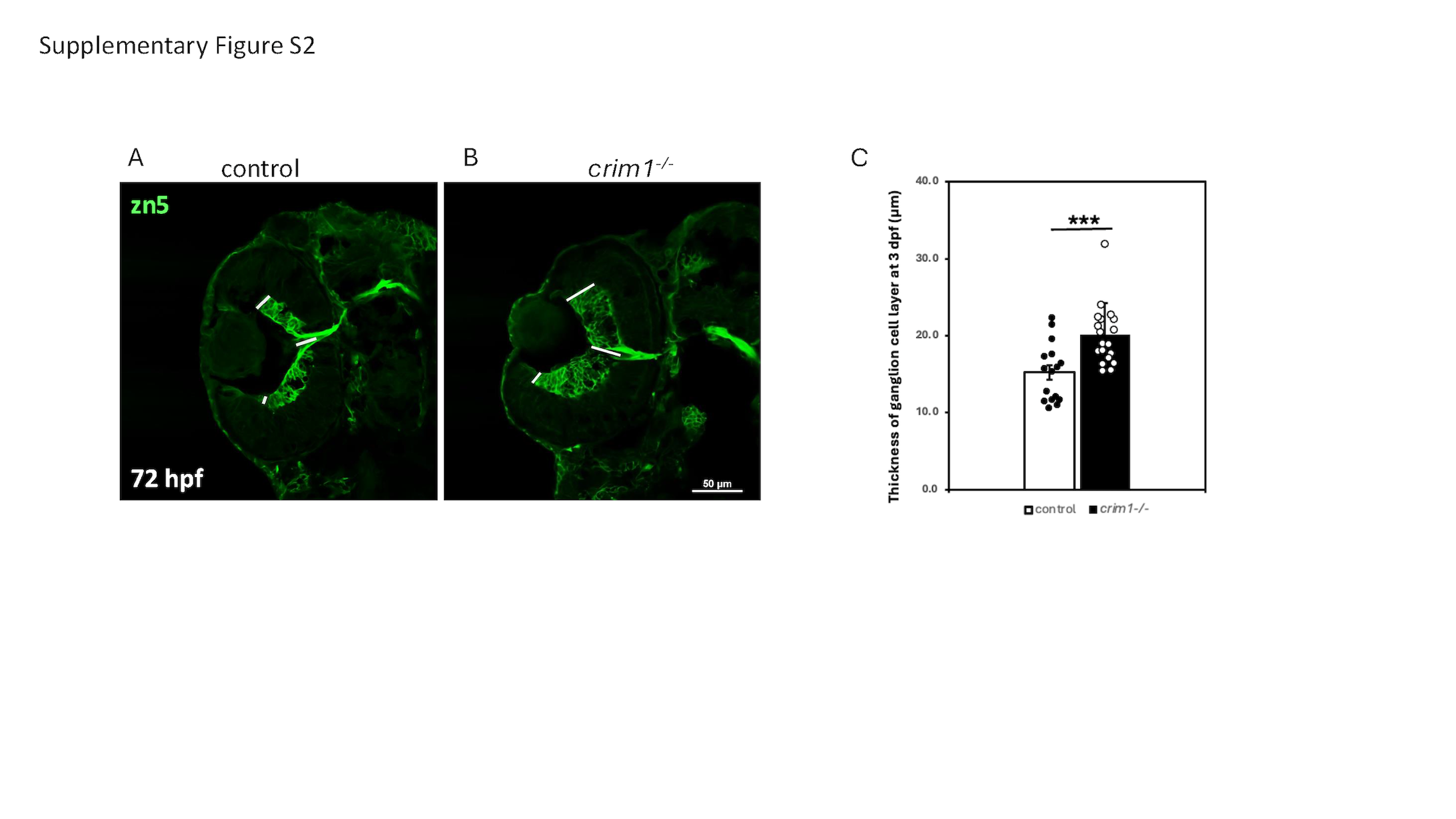

Supplement: Supplementary file 13 [file Image2.tiff]

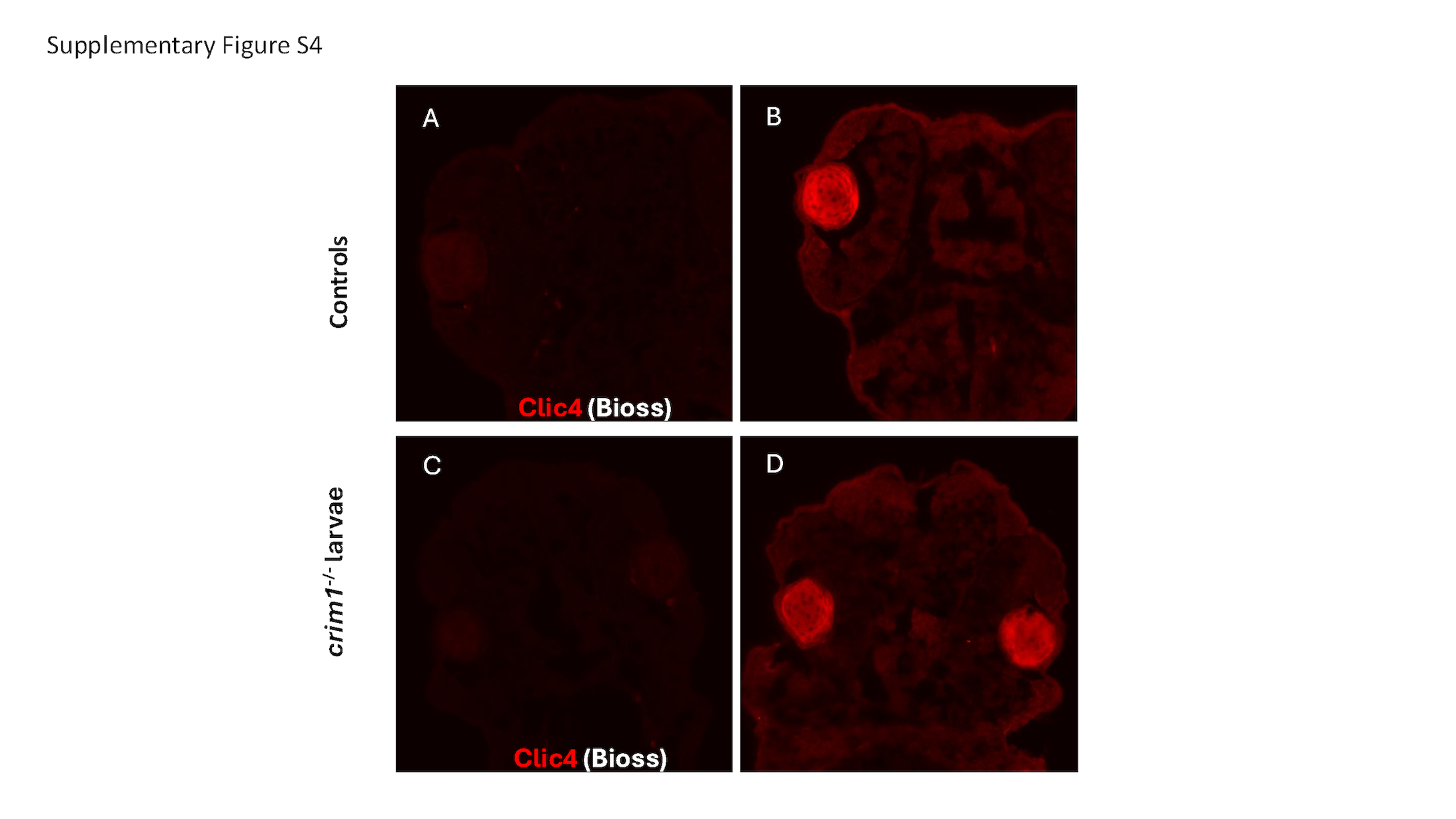

Supplement: Supplementary file 14 [file Image4.tiff]

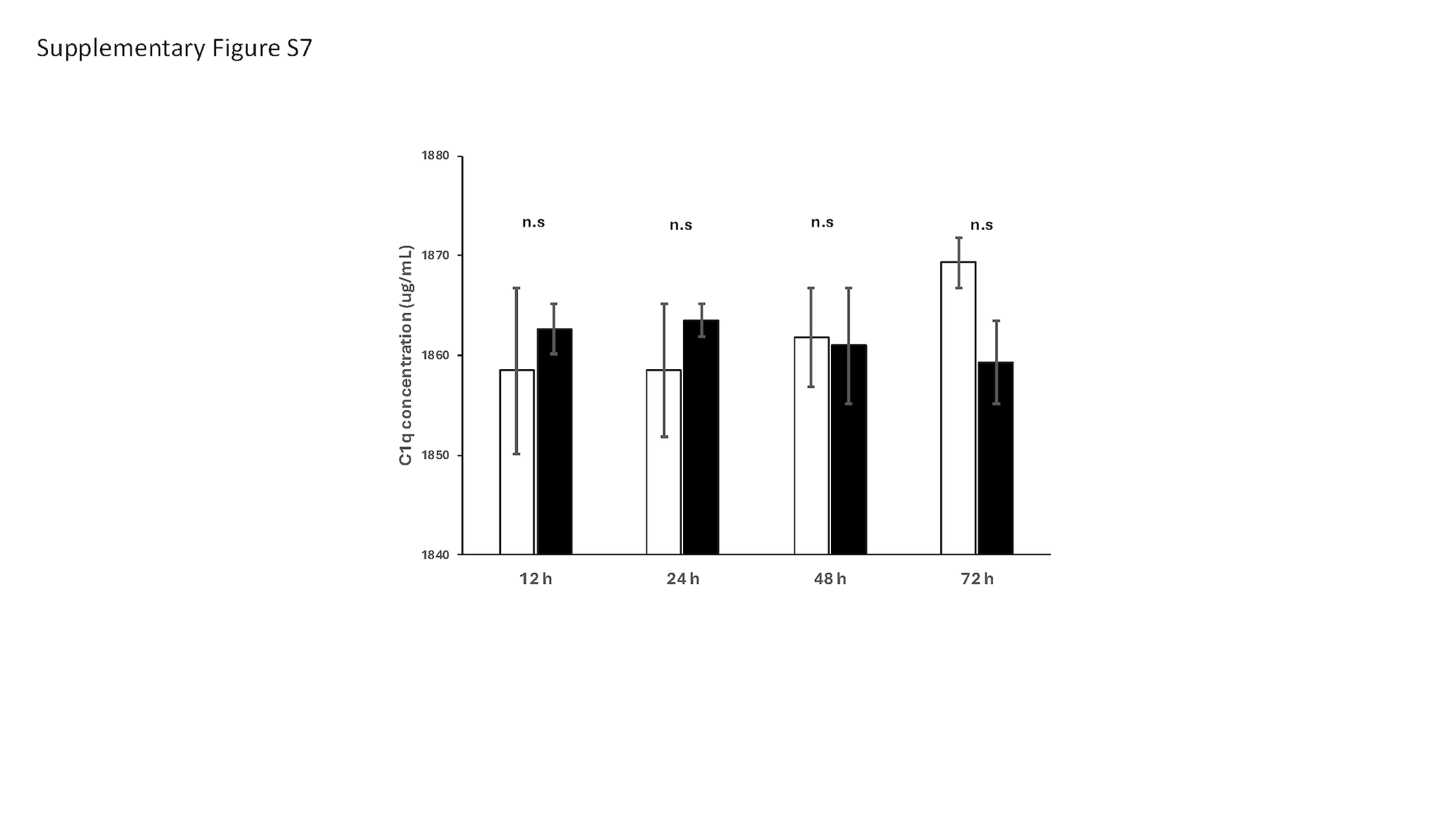

Supplement: Supplementary file 17 [file Image7.tiff]
