# Supplementary material for: A zebrafish model of crim1 loss of function has small and misshapen lenses with dysregulated clic4 and fgf1b expression
Source: Front Cell Dev Biol. 2025 Mar 6;13:1522094. doi: 10.3389/fcell.2025.1522094 (PMC11922885; doi:10.3389/fcell.2025.1522094)
Supplement: Supplementary file 4 [file Table1.docx]

**Supplementary Table S1. Ocular and extraocular findings in murine models of altered *Crim1* function**

| **Mouse model** | **Mechanism** | **Ocular findings** | **Extraocular findings** | **References** |
| --- | --- | --- | --- | --- |
| *Crim1*^KST264/KST264^ | Hypomorph or dominant negative allele | Reduced lens size; accumulation of cells with endothelial appearance in posterior eye chamber; restricted aperture of the pigmented anterior optic cup | Syndactyly of digits 3 and 4 of forelimbs and hindlimbs; transient blebbing of skin on midline of head; placental hypoplasia; cerebral edema; reduced renal size; placental defects; perinatal lethal on a B6 background and survived to adulthood on a mixed background | Pennisi et al., 2007;  Pennisi et al., 2012 |
| *Crim1*^KST264/KST264^ | Hypomorph or dominant negative allele | - | Glomerular defects with leaky peritubular vasculature; progressive interstitial fibrosis; hydronephrosis; glomerular cysts | Wilkinson et al., 2007; Wilkinson et al., 2009; Wilkinson et al., 2012  Bottom of Form |
| Crim1^Δflox/Δflox^ | Hypomorph or dominant negative allele | Eye hypoplasia; eye dysgenesis | Mild digit syndactyly; peridermal blebbing; renal hypoplasia, glomerular dysgenesis; widespread edema | Chiu et al. 2012 |
| Crim1^Δflox/Δflox^ | Hypomorph or dominant negative allele; Conditional Crim1 KO^1^ mice in vascular  endothelial cells | Eye hypoplasia; defective retinal vascular development | Syndactyly; peridermal blebbing; edema; hemorrhage | Fan et al., 2014 |
| *Crim1*^flox/flox^ Ap2α-cre;  Homozygous | Hypomorph or dominant negative allele in head surface ectoderm/ocular mesenchyme | Small, pear-shaped lenses with deficient development of the lens fiber cell mass; microcornea; shallow anterior chamber; narrower eye without diminished axial diameter | Perinatal lethality | Beleggia et al., 2015 |
| *Crim1*^glcr11/glcr11^ | Hypomorphic allele; functionally truncated protein | Smaller. misshapen lenses; reduced proliferation and numbers of LE^1^ cells; disorganized LE cell-cell adhesions; posterior lens cataract; retinal dysplasia; microphthalmia | - | Zhang et al., 2016 |
| *Crim1*^KST264/KST264^ | Hypomorphic or gain of function allele | Microphthalmia; small lenses; anterior extension of the optic cup margin; reduced anterior opening of the optic cup; Increased number of hyaloid cells in posterior vitreal chamber; narrow zone of LE^2^ cell proliferation; early differentiation of LE cells to LF^3^ cells; aphakia | Glomerular cysts; loss of endothelial integrity, progressing to peritubular and pericystic fibrosis; detachment of peritubular capillary endothelial cells from the basement membrane | Phua et al. 2013;  Fan et al., 2014;  Tam et al., 2018 |
| *Crim1*^C140S/C140S^ | Hypomorphic allele | Microphthalmia | Dwarfism; enlarged seminal vesicles; rectal prolapse; syndactyly of the hindlimbs; renal atrophy; small body size; kyphosis; perinatal lethal on a B6 background and survived to adulthood on a mixed background | Furuichi et al., 2019 |

KO^1^ = knock-out; LE^2^ = lens epithelial cells; LF^3^ = lens fiber cells.
