# Supplementary material for: A zebrafish model of crim1 loss of function has small and misshapen lenses with dysregulated clic4 and fgf1b expression
Source: Front Cell Dev Biol. 2025 Mar 6;13:1522094. doi: 10.3389/fcell.2025.1522094 (PMC11922885; doi:10.3389/fcell.2025.1522094)
Supplement: Supplementary file 8 [file Table2.docx]

**Supplementary Table S2. Ocular and extraocular findings in zebrafish models of altered *crim1* function**

| **Zebrafish model** | **Mechanism** | **Ocular phenotype** | **Extraocular phenotype** | **References** |
| --- | --- | --- | --- | --- |
| Antisense morpholinos (MOs) targeting translational start site and 5’UTR | Loss of function | Small eyes | Bent or hooked or twisted tails with U-shaped somites; smaller heads; notochord constriction; loss of muscle pioneer cells; expansion of the ventral mesoderm-derived intermediate cell mass; irregular or absent blood circulation with defective blood vessels | Kinna et al., 2006 |
| Antisense MOs targeting translational start site | Loss of function | Microphthalmia; low dose of crim1 MO at 1.0-1.5 ng - increased nuclei clustered in the medial portion of the lens; high dose of crim1 MO at 2.5-3.0 ng- greater increase of nuclei in lens coupled with microphthalmia and defective lamination; low-dose crim1 knockdown induces mild form of cataracts | NA | Brastrom et al., 2019 |
| *crim1* homozygous 2 basepair deletion | Hypomorphic allele | Small lenses; microphthalmia | NA | This paper |
