# Supplementary material for: A zebrafish model of crim1 loss of function has small and misshapen lenses with dysregulated clic4 and fgf1b expression
Source: Front Cell Dev Biol. 2025 Mar 6;13:1522094. doi: 10.3389/fcell.2025.1522094 (PMC11922885; doi:10.3389/fcell.2025.1522094)
Supplement: Supplementary file 9 [file Table5.docx]

**Supplementary Table S5. Primers used for quantitative reverse transcriptase polymerase chain reaction (RT-qPCR)**

| **Gene** | **Transcript** | **Forward primer** | **Reverse primer** | **Product size** |
| --- | --- | --- | --- | --- |
| *bzw1a* | NM_199708.1 | 5’-TCTGCATTCATTTCGGTGTCTT-3’ | 5’-GTGCCAGTTTGGTTCAAGCC-3’ | 158 bp |
| *crim1* | NM_212821.1 | 5’-AGGACGTGCGCCTGTAAGAG-3’ | 5’-ACGACACTGGCAAACATCGC-3’ | 120 bp |
| *clic4* | NM_201486.1 | 5’-TCTTCGCGCTTGTAGGCATT-3’ | 5’-GCCTCCACACGTATGTTCCT-3’ | 170 bp |
| *fgf1b* | NM_001105278.1 | 5’-GTTGTGGCCATCAGAGGTCAT-3’ | 5’-ACCAGTCTCCGTTGTCCTGAT-3’ | 169 bp |
| *gpib* | NM_144764.2 | 5’-TAAAGTCCGCAGTGGCGAGT-3’ | 5’-AGCCTCGGTCACCATTAGCG-3’ | 109 bp |
| *itgb1* | NM_001034987.1 | 5’-ACCTCATTCCCAAGTCAGCAGT-3’ | 5’-TTGCAGTGGGAGACGTAGGA-3’ | 157 bp |
| *nampta* | XM_002661340.6 | 5’-GGTGCTTCCGCCGTACATTC-3’ | 5’-CTCCTTCATGCCCTCCACGA-3’ | 85 bp |
| *serpin1l2* | NM_001045171.2 | 5’-AGCAGATGACTCTCGCCAGTT-‘3 | 5’-CTGGGATGCATCTGTAGGGAA-3’ | 260 bp |
| *zgc:153846* | NM_001076649.1 | 5’-TCCTGCCGCATGATTCCTTCA-3’ | 5’-GCCAGTGACCGTCCATCACAT-3’ | 166 bp |
| *eef1a1l1* | NM_131263.1 | 5’-GGAGACTGGTGTCCTCAA-3’ | 5’-GGTGCATCTCAACAGACTT-3’ | 86 bp |
