# Supplementary material for: A zebrafish model of crim1 loss of function has small and misshapen lenses with dysregulated clic4 and fgf1b expression
Source: Front Cell Dev Biol. 2025 Mar 6;13:1522094. doi: 10.3389/fcell.2025.1522094 (PMC11922885; doi:10.3389/fcell.2025.1522094)
Supplement: Supplementary file 10 [file Table3.docx]

**Supplementary Table S3. Clustered Regularly Interspaced Short Palindromic Repeats (CRISPR)/** **CRISPR-associated protein 9** (**Cas9) target site and amplification primers**

| Target site | Forward primer | Reverse primer |
| --- | --- | --- |
| GGGCTTTGAGCCGT | 5’-TGCTTGTGTTTAATCTGGCGA-3’ | 5’-CAATCTTCTGTAGCGCCGTC-3’ |
