# Supplementary material for: A zebrafish model of crim1 loss of function has small and misshapen lenses with dysregulated clic4 and fgf1b expression
Source: Front Cell Dev Biol. 2025 Mar 6;13:1522094. doi: 10.3389/fcell.2025.1522094 (PMC11922885; doi:10.3389/fcell.2025.1522094)
Supplement: Supplementary file 11 [file Table4.docx]

**Supplementary Table S4. Antibodies used in the study: Details and sources**

| **Protein expression by IHC/ Western blotting** | **Primary antibody** | **Concen-tration** | **Secondary antibody** | **Concen-tration** | **Source** |
| --- | --- | --- | --- | --- | --- |
| ***IHC*** |  |  |  |  |  |
| zl-1 | Mouse anti-zl-1 | 1 in 500^1^ | Donkey anti mouse IgG A488, A21202 | 1 in 400 | ZIRC^2^, Eugene, OR |
| zn-5 | Mouse anti-zn-5 | 1 in 20 | Donkey anti mouse IgG A488, A21202 | 1 in 400 | ZIRC, Eugene, OR |
| zpr1 | Mouse anti-zpr1 | 1 in 20 | Donkey anti mouse IgG A488, A21202 | 1 in 400 | ZIRC, Eugene, OR |
| E-cadherin | Mouse anti-E-cadherin | 1 in 100 | Donkey anti mouse IgG A488, A21202 |  | 610181; BD BioSciences, San Jose, CA |
| clic4 | Rabbit anti-clic4 | 1 in 100 | Goat anti rabbit IgG A488,  A11008 | 1 in 400 | bs-7098R; Bioss Antibodies, Woburn, MA |
| crim1 | Rabbit anti-crim1 | 1 in 100 | Goat anti rabbit IgG A488,  A11008 | 1 in 100 | bs-2034R; Bioss Antibodies, Woburn, MA |
| fgf1 | Mouse anti-fgf1 | 1 in 100 | Donkey anti mouse IgG A488, A21202 | 1 in 400 | sc-55522; Santa Cruz Biotechnology, Dallas, TX |
| cleaved *caspase-3* | Rabbit anti-caspase3 | 1 in 500 | Goat anti rabbit IgG A488,  A11008 | 1 in 400 | 9664; Cell Signaling Technology, Danvers, MA |
| ***Western blotting*** |  |  |  |  |  |
| Crim1 | Rabbit anti-crim1 (polyclonal) | 1 in 100-1 in 500 | See footnote^2^ | See footnote^2^ | bs-2034R; Bioss Antibodies, Woburn, MA |
| Clic4 | Rabbit anti-clic4 (polyclonal) | 1 in 1,000 |  |  | 12298-2-AP; Proteintech, Rosemont, IL |
| Clic4 | Rabbit anti-clic4  (polyclonal) | 1 in 100 |  |  | bs-7098R; Bioss Antibodies, Woburn, MA |
| Clic4 | Rabbit anti-clic4 (monoclonal) | 1 in 10,000 |  |  | ab183043; Abcam, Waltham, MA |
| Itgb1 | Rabbit anti-itgb1 (monoclonal) | 1 in 100 |  |  | MA531964; Invitrogen, Carlsbad, CA |
| Fgf1b | Mouse anti-fgf1b(monoclonal) | 1 in 100 |  |  | sc-55520; Santa Cruz Biotechnology, Dallas, TX |
| Gapdh | Mouse anti-gapdh (monoclonal) | 1 in 2,000 |  |  | TA802519; Origene, Rockville, MD |

1 in 500^1^ = see Zebrafish International Resource Center (ZIRC) site for recommended antibody concentrations. See footnote^2^ = Secondary antibodies included goat anti-rabbit IRDye 680, goat anti-rat IRDye 680, and donkey anti-mouse IRDye 680 (1:20,000, LI-COR, Lincoln, NE, USA).
