# Supplementary material for: A zebrafish model of crim1 loss of function has small and misshapen lenses with dysregulated clic4 and fgf1b expression
Source: Front Cell Dev Biol. 2025 Mar 6;13:1522094. doi: 10.3389/fcell.2025.1522094 (PMC11922885; doi:10.3389/fcell.2025.1522094)
Supplement: Supplementary file 16 [file DataSheet1.docx]

**Supplementary data**

**Materials and Methods**

*Immunohistochemistry*

Embryos were fixed in 4% paraformaldehyde/phosphate buffered saline (PBS) overnight at 4°C. They were incubated in 10% sucrose/PBS, %, 25% sucrose/PBS, and 35% sucrose/PBS volume:volume and then embedded in Tissue-Tek® O.C.T compound (Sakura-Finetek, Torrance, CA, USA), frozen on dry ice, and stored at -80°C. 8 μm tissue sections were cut and rehydrated with 1XPBS, blocked in 5% goat serum (Thermo Fisher Scientific, Waltham, MA) in 1xPBS containing 1% bovine serum albumin (BSA), 0.3% TritonX100 and 0.1% Tween®20 at room temperature (RT). Slides were incubated with primary antibody overnight at 4°C. The appropriate rabbit anti-primary antibody and mouse anti primary antibody conjugated with anti-rabbit Alexa Fluor 488 and anti-mouse Alexa Fluor 488 (Invitrogen, Grand Island, New York) were used at a dilution of 1:400 for 1 hr at RT. Primary antibodies were: zl-1 (1:500), zn-5 (1:20), zpr-1 (1:20; Zebrafish International Resource Center, Eugene, OR), E-Cadherin (#610181, 1:100; BD BioSciences, San Jose, CA), clic4 (#bs-7098R, 1:100; Bioss Antibodies, Woburn, MA), crim1 (#bs-2034R, 1:100; Bioss Antibodies, Woburn, MA), fgf-1 (sc-55522, 1:100; Santa Cruz Biotechnology, Dallas, TX), cleaved caspase-3 (#9664, 1:500; Cell Signaling Technology, Danvers, MA). Sections were counterstained with 1:1000 DAPI (10mg/mL; MilliPore-Sigma, Norwood, OH) for 5 minutes at RT, washed 3x with PBS, and mounted with FluorSave Reagent (#345789; MilliPore-Sigma, Norwood, OH). For H & E staining, frozen sections through the optic nerve were collected, stained with H & E (Sigma-Aldrich, St. Louis, MO). Images were obtained with 40x and 60x objectives on a **Nikon Ni-E upright wide-field microscope and**a Nikon Eclipse Ti2-E inverted Motorized confocal microscope (Nikon, Japan). Images of live embryos were captured with a Zeiss optical SteREO bright field microscope (Zeiss, Oberkochen, Germany). Image view and the measurements were performed using ImageJ software (National Institutes of Health, MD). Antibodies have been listed in Supplementary Table S4.
